# Cardiovascular Mettl3 Deficiency Causes Congenital Cardiac Defects and Postnatal Lethality in Mice

**DOI:** 10.7150/ijbs.100941

**Published:** 2025-03-10

**Authors:** Qianqian Feng, Lihua Qi, Jiaqi Huang, Zhigang Dong, Fang Yu, Jing Zhang, Jun Zhan, Hongquan Zhang, Wengong Wang, Yong Zhou, Zhongzhou Yang, Yuan Zhou, Wei Kong, Yi Fu

**Affiliations:** 1 Department of Physiology and Pathophysiology, School of Basic Medical Sciences, Peking University; State Key Laboratory of Vascular Homeostasis and Remodeling, Beijing 100191, China.; 2 Program for Cancer and Cell Biology, Department of Human Anatomy, Histology and Embryology, School of Basic Medical Sciences, Peking University Health Science Center, Beijing 100191, China.; 3 Department of Biochemistry and Molecular Biology, Beijing Key Laboratory of Protein Posttranslational Modifications and Cell Function, School of Basic Medical Sciences, Peking University Health Science Center, 38 Xueyuan Road, Beijing 100191, China.; 4 CAS Key Laboratory of Tissue Microenvironment and Tumors, Shanghai Institute of Nutrition and Health, Shanghai Institutes for Biological Sciences, Chinese Academy of Sciences, Shanghai 200031, China.; 5 State Key Laboratory of Pharmaceutical Biotechnology, MOE Key Laboratory of Model Animal for Disease Study, Model Animal Research Center, and Jiangsu Key Laboratory of Molecular Medicine, Nanjing University Medical School, Nanjing 210093, China.

**Keywords:** METTL3, m^6^A RNA methylation, embryonic heart development, congenital heart disease

## Abstract

N^6^-methyladenosine (m^6^A) is the most common epigenetic modification of RNA, but whether m^6^A RNA methylation modulates cardiovascular development or congenital heart diseases (CHDs) has not been determined. The published high-throughput sequencing data suggested that transcripts of genes related to CHDs were prone to m^6^A modification, while the expression of methyltransferase-like 3 (METTL3)-involved methyltransferase complex was downregulated in mouse embryonic hearts following prenatal alcohol exposure as a critical CHD risk factor, indicating the association of insufficient m^6^A RNA methylation with CHDs. Using cardiovascular-specific *Mettl3* knockout mice (*Tagln-Cre*; *Mettl3^flox/flox^*), we observed that cardiovascular *Mettl3* deficiency resulted in postnatal lethality and profound congenital cardiac defects, including left pulmonary stenosis, ventricular septal defects, and right ventricular hypoplasia. The m^6^A-specific methylated RNA-immunoprecipitation sequencing identified *Sox4*, *Sox11*, and *Mef2a*, the critical transcription factors involved in the right ventricle and outflow tract development, were the regulatory targets of METTL3-catalyzed m^6^A RNA methylation. *Mettl3* deficiency-caused insufficient m^6^A RNA methylation downregulated the expression of SOX4, SOX11, and MEF2A in mouse embryonic hearts. In conclusion, cardiovascular *Mettl3* deficiency directly led to congenital cardiac defects by downregulating the m^6^A-dependent expression of *Mef2a*, *Sox4*, and *Sox11*. METTL3-catalyzed m^6^A RNA methylation may become a potential target for preventing and treating CHDs.

## Introduction

Congenital heart disease (CHD) is the most common human birth anomaly and is life-threatening. CHDs encompass a broad spectrum of cardiac defects ranging from a solitary abnormality of the ventricular septum, semilunar valves, or outflow tract to complex lesions of multiple defects (e.g., tetralogy of Fallot or hypoplastic left heart syndrome). Through extensive genome-wide association studies or whole-exon sequencing in patients, more than 400 genes are associated with the etiology of congenital cardiac defects 1. These genes encode cardiac development-related transcription factors (e.g.,* GATA4* 2, *NKX2.5* 3, *HAND2* 4, and *TBX5* 5) and morphogen signaling molecules (e.g., *BMP4* 6, *JAG* 7, and *FLT4* 8), as well as heart structure components (e.g., *MYBPC3* 9, *GJA5*, and *MYH6* 10). Nevertheless, only one-third of CHDs are attributed to genetic factors 11. Epigenetic regulation is an extra-genomic mechanism that does not involve alterations in the DNA sequence but is capable of regulating gene expression by influencing transcription or translation. Epigenetic modifications, such as DNA methylation, histone modification, and ATP-dependent chromatin remodeling, are also significantly associated with the pathogenesis of CHDs 12-15. However, the current understanding of the regulatory mechanisms of congenital cardiac defects, particularly epigenetic modifications, is still limited.

N^6^-methyladenosine (m^6^A) is the most abundant epigenetic modification of RNA in eukaryotes and modulates gene expression by affecting the fate of target RNA 16, 17. Methyltransferase-like 3 (METTL3), which functions as the core catalytic subunit, combines with METTL14, Wilms' tumor 1-associating protein (WTAP), and other regulatory subunits to form the m^6^A methyltransferase complex and mediate m^6^A RNA methylation, while demethylases alkB homolog 5 (ALKBH5) and fat mass and obesity-associated protein (FTO) remove the m^6^A RNA methylation 18, 19. Therefore, *Mettl3* deficiency directly causes dysfunctional m^6^A RNA methylation. Global knockout of *Mettl3* causes embryonic lethality in mice, indicating that METTL3-catalyzed m^6^A RNA methylation plays a critical role in embryonic development in mice 20, whereas ALKBH5 overexpression in embryonic stem cells reduced the level of m^6^A and substantially impaired cardiomyocyte differentiation and proliferation *in vitro* 21. To date, m^6^A RNA methylation has been reported to regulate the development of various organs/systems, such as the liver, bone, hematopoietic system, central nervous system, and reproductive system 22-26. Although several *in vitro* studies have shown increased m^6^A RNA methylation during the differentiation of embryonic stem cells into cardiomyocytes 21, whether m^6^A RNA methylation modulates cardiovascular development or CHDs has not been determined.

Here, we found that cardiovascular *Mettl3* deficiency resulted in postnatal lethality and congenital cardiac defects in mice, including left pulmonary stenosis, ventricular septal defects, and right ventricular hypoplasia. Mechanistically, METTL3 catalyzes the formation of m^6^A RNA methylation on transcripts of* Sox11*, *Sox4*, and *Mef2a*, which are the critical transcription factors involved in cardiovascular development, thus facilitating the expression of these proteins. Thus, METTL3-catalyzed m^6^A RNA methylation may be a potential modulatory target for CHD intervention.

## Materials and Methods

### Materials

Antibodies against METTL3 (ab195352), SOX4 (ab86809), SOX11 (ab134107), and MEF2A (ab109420) used for Western blotting analysis and/or immunohistochemistry staining were purchased from Abcam (Cambridge, UK). Antibodies against MEF2A (sc-17785) and PCNA (sc-56) used for Western blotting analysis were purchased from Santa Cruz Biotechnology (Texas, USA). Antibodies against GATA5 (55433-1-AP) and GAPDH (10494-1-AP) used for Western blotting analysis were purchased from Proteintech Group, Inc. (Wuhan, China). Antibodies against Cyclin D1 (2978) and the normal rabbit IgG (2729) used for Western blotting analysis or MeRIP-qPCR assay were purchased from Cell Signaling Technology (Boston, USA). The antibody against m^6^A (202003) used for the MeRIP-qPCR assay was purchased from Synaptic Systems (Goettingen, DE). IRDye-conjugated secondary antibodies used for Western blotting analysis were purchased from Rockland, Inc. (Gilbertsville, USA). A hematoxylin and eosin staining kit (C0105S) was purchased from Beyotime Biotechnology (Shanghai, China). HRP-conjugated secondary antibodies immunohistochemistry kit (PV6001) for immunohistochemistry staining was purchased from ZSGB-BIO (Beijing, China).

## Methods

### Animals and prenatal alcohol exposure

The animal studies were approved by the Biomedical Ethics Committee of Peking University (No. DLASBD0092). All animal-related procedures conformed to the guidelines of the Institutional Animal Care and Use Committee of Peking University Health Science Center. The mice were maintained in groups and kept on a 12-hour light-dark cycle, with free access to food and water, and the room temperature was maintained at about 22°C. For tissue isolations, mice were anesthetized using isoflurane (2%, inhalation). The mice were euthanized by cervical dislocation under isoflurane anesthesia.

*Mettl3^flox/flox^* mice constructed by Biocytogen Pharmaceuticals (Beijing) Co., Ltd. were kindly provided by Prof. Zengqiang Yuan from the Beijing Institute of Basic Medical Sciences. Briefly, two LoxP sites were inserted into both ends of exon 2 and exon 3 of the mouse *Mettl3* gene by using the CRISPR/Cas9 system 27. Genotyping was performed by PCR using the primers listed in Table S1. The length of the PCR product is 443 bp (flox) or 351 bp (wildtype).

*Tagln-Cre* mice were purchased from the Jackson Laboratory (Strain #:017491). Genotyping was performed by PCR using the primers in Table S1. The length of the *Cre^+^* PCR product is about 300 bp and the length of the internal reference PCR product is about 200 bp.

To generate cardiovascular-specific *Mettl3* knockout mice, female *Mettl3^flox/flox^* mice were crossed with male transgenic mice expressing Cre recombinase under the control of the *Tagln* gene promoter. Both *Mettl3^flox/flox^* and *Tagln-Cre* mice were maintained with a C57BL/6 strain background. This breeding strategy, as indicated in Figure 3B, yielded an expected 25% of the progeny with *Mettl3^flox/flox^* mice as control littermates and 25% *Tagln-Cre*;* Mettl3^flox/flox^* mice were cardiovascular-specific *Mettl3* knockout mice (also called *Mettl3*-CV KO). Embryonic day (E) 0.5 was defined as the day at which the vaginal plug was detected. The mice within 24 hours after birth were defined as postnatal day (P) 0. The ratio of the diameter of the left pulmonary artery (LPA) to the diameter of the descending aorta (DA) in the same mouse was determined to evaluate pulmonary stenosis 28, 29. The diameters of the LPA and DA were directly measured in dissected tissues *ex vivo*.

As previously reported 30, 31, two doses of 25% ethanol in saline (vol/vol) or vehicle alone at 0.015 mL/g body weight were injected into pregnant females intraperitoneally each day on E8.5 and E9.5. The pregnant mice were euthanized on E10.5 or E18.5 and the embryonic hearts were collected.

### Bioinformatics analysis of public sequencing data

Cardiac m^6^A RNA methylation target genes were identified by analysis of a public MeRIP sequencing dataset from neonatal rat hearts (GSE162545). Briefly, the raw FASTQ reads were downloaded, and only the data from day 0 after delivery were retained. Quality control, low-quality filtration, and read de-adaptor functions were performed with the fastp tool to obtain clean reads. The clean reads were subsequently aligned to the rat reference genome (rn7, with gene annotations from the Ensembl database v108) via HISAT2 software. Finally, the m^6^A RNA methylation peaks were identified by using the exomePeak2 32, 33 R package (https://bioconductor.org/packages/release/bioc/html/exomePeak2.html), and the genes harboring at least one significant m^6^A peak were deemed cardiac m^6^A RNA methylation target genes. Subsequently, we determined the relative enrichment of cardiac m^6^A RNA methylation target genes among CHD-related genes by the chi-square test. The genes that are known to be associated with the generic CHD terms and more specific terms, including tetralogy of Fallot (TOF), ventricular septal defects (VSD), pulmonary stenosis (PS), patent ductus arteriosus (PDA), atrial septal defects (ASD), and atrioventricular septal defects (AVSD), were obtained from DisGeNET (https://www.disgenet.org/search), a comprehensive database of disease-related genes 34.

The mouse embryonic development spatiotemporal transcriptome atlas (MOSTA, https://db.cngb.org/stomics/mosta/) was generated as described previously 35. According to generic CHD terms obtained from the DisGeNET database, we acquired gene expression patterns from the MOSTA website.

### Hematoxylin-eosin (HE) and immunohistochemistry (IHC) staining

The tissues were fixed with 4% paraformaldehyde overnight at 4°C and embedded in paraffin. Serial sections (6 μm) were stained with hematoxylin and eosin for histopathological analysis. Immunohistochemical staining was performed on 6-µm-thick paraffin sections using primary antibodies and HRP-conjugated secondary antibodies immunohistochemistry kit (PV6001) from ZSGB-BIO according to the manufacturer's instructions. The staining images were captured using a Nano Zoomer inverted microscope.

### m^6^A-specific methylated RNA immunoprecipitation (MeRIP)

Total RNA was extracted from the hearts of E10.5-11.5 *Mettl3*-CV KO embryos or control littermates using TRIzol reagent (Invitrogen, Carlsbad, CA, USA), and 100 μg of total RNA was used for each group of MeRIP experiments. Total RNA was fragmented into approximately 200 nt fragments using RNA fragmentation reagents (AM8740, Invitrogen) according to the manufacturer's instructions and subsequently reprecipitated with one-tenth the volume of 3 mol/L sodium acetate in a 2.5 volume of 100% ethanol at -80°C overnight. After redissolution, one-tenth of the RNA fragments were saved as "input", and the remaining fragments were subjected to m^6^A immunoprecipitation. The RNA was incubated with 5 μg of anti-m^6^A antibody (202003, Synaptic Systems) or rabbit IgG with RNase inhibitors in 1× immunoprecipitation buffer (150 mmol/L NaCl, 10 mmol/L Tris, pH = 7.4) at 4°C overnight with rotation. Then, 60 µl of Pierce™ Protein A/G Magnetic Beads (88803, Thermo Scientific) was prewashed and mixed with antibody-conjugated RNA at 4°C for 4 hours with rotation. After 5 washes with 1× immunoprecipitation buffer, the combined RNA samples were extracted using TRIzol reagent and precipitated with GlycoBlue Coprecipitant (AM9515, Invitrogen) in isopropanol at -20°C overnight. Further enrichment was calculated by reverse transcription-qPCR, and the corresponding m^6^A enrichment in each sample was calculated by normalization to the input.

### Nano UMI MeRIP-sequencing and analysis

The Nano UMI MeRIP experiment, high-throughput sequencing, and data analysis were conducted by Seqhealth Technology Co., Ltd. (Wuhan, China). The right ventricle and outflow tract tissues from E10.5-11.5 *Mettl3*-CV KO and control embryos were isolated. Each group consisted of two biological duplicate samples and each sample contained tissues from 20 embryos. The RNA was extracted using the TRIzol reagent. Total RNA (5 μg) was fragmented into approximately 200 nt fragments and used for the MeRIP experiment. A specific anti-m^6^A antibody (Synaptic Systems, 202003) was used for m^6^A immunoprecipitation. The stranded RNA sequencing library was constructed by using the KC-Digital^TM^ Stranded mRNA Library Prep Kit for Illumina® (Catalog NO. DR08502, Wuhan Seqhealth Co., Ltd., China) with five PCR cycles. The kit eliminates duplication bias in PCR and sequencing steps by using a unique molecular identifier (UMI) of 8 random bases to label the preamplified cDNA molecules. Then, ribosomal cDNA was removed with a SMARTer Stranded Total RNA-Seq Kit version 2 (Pico Input Mammalian, 634413, Takara/Clontech, Japan), and ten PCR cycles were added. The library products of 200-500 bp were enriched, quantified, and finally sequenced on a PE150 model DNBSEQ-T7 sequencer (MGI Tech Co., Ltd., China). Quality control, low-quality filtering, and read de-adaptor functions were performed with the fastp tool to obtain clean reads. The clean reads were subsequently aligned to the rat reference genome (mm39, with gene annotations from the Ensembl database v108) via HISAT2 software. Finally, the m^6^A RNA methylation peaks were identified by using the exomePeak2 R package. The datasets produced in this study are available in Gene Expression Omnibus databases (GSE288034).

### Quantitative real-time PCR (qPCR)

Total RNA was extracted from the cardiac tissues of *Mettl3*-CV KO embryos or control littermates using TRIzol reagent (Invitrogen) and quantified. No more than 1 μg of extracted RNA was utilized as a template for reverse transcription after the enzymatic digestion of genomic DNA with a NovoScript® II Reverse Transcriptase Kit (Novoprotein, Shanghai, China). qPCR was performed on a Thermo Fisher QuantStudio 3 instrument, and the results were analyzed with QuantStudio™ Design & Analysis Software. The mRNA expression was normalized to that of GAPDH. The primers are listed in Table S2.

### Protein extraction and Western blotting

The entire hearts of *Mettl3*-CV KO embryos or control littermates were dissected under a stereoscope. Tissues were ultrasonically crushed and lysed in RIPA buffer (Beyotime) supplemented with 1× proteinase inhibitor cocktail and 1% PMSF on ice. Following centrifugation, the cleared lysates were quantified by a BCA assay and then loaded on a 10-12% SDS‒PAGE gel. After gel electrophoresis separation, the proteins were transferred to nitrocellulose membranes, blocked with 5% BSA-TBST, and incubated with primary antibodies at 4°C overnight. After washing, the membranes were incubated with secondary antibodies. Images were captured by LI-COR Odyssey (9120) imaging system, and band densities were quantified using ImageQuant TL software (GE). The protein expression was normalized to that of GAPDH.

### Statistical analysis

All the quantitative data are presented as the mean ± SEM. Statistical analysis was performed using GraphPad Prism 8.0 software (GraphPad Software, San Diego, CA, USA). For normally distributed data, a Student's *t* test was applied for two-group comparisons, while ordinary ANOVA was used for comparisons of more than two groups where equal variances were assumed. In addition, the chi-square (and Fisher's exact) test and the Gehan-Breslow-Wilcoxon test were also utilized for specific analyses, such as incidence/fraction comparisons and Kaplan‒Meier survival curve data. A *P* value < 0.05 was regarded as statistically significant. The detailed statistical analyses used for each experiment are presented in the corresponding figure legends.

## Results

### METTL3-catalyzed m^6^A RNA methylation is related to congenital heart disease

**Figure 1:**
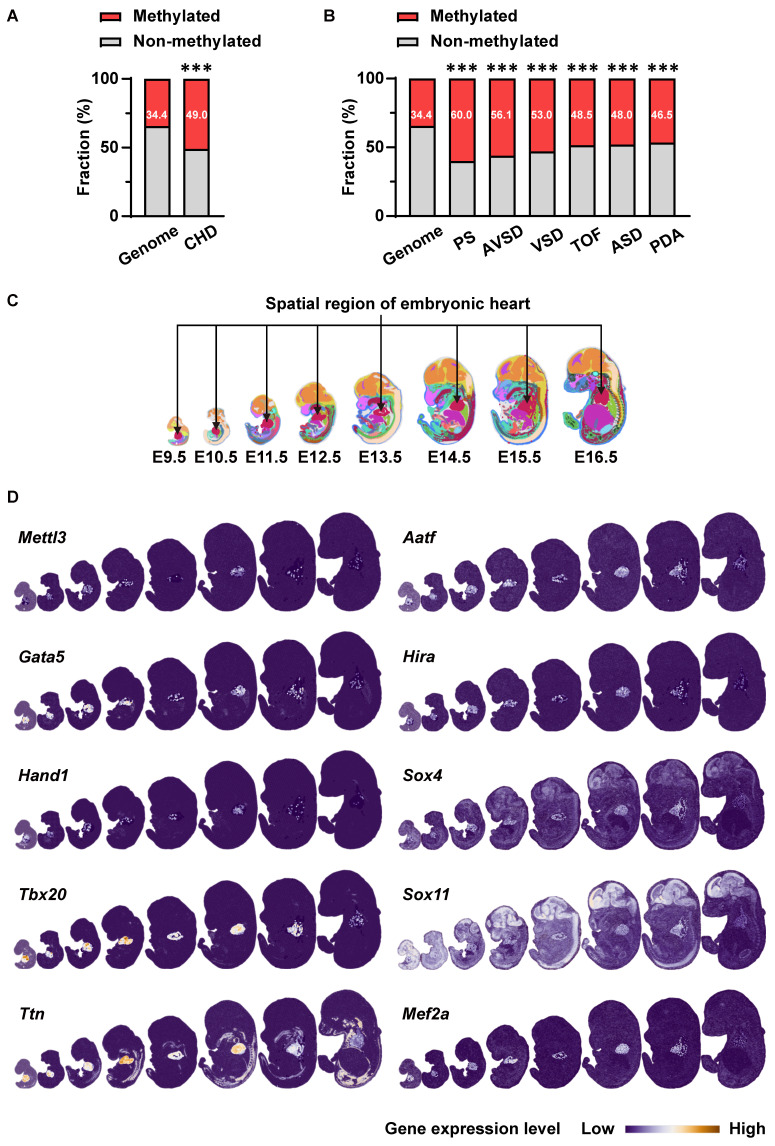
** METTL3-catalyzed m^6^A RNA methylation is involved in embryonic cardiac development. A** The proportions of m^6^A methylation target genes in neonatal rat hearts acquired from Gene Expression Omnibus (GEO) data (GSE162545) among all coding genes (genome, n = 21657 genes) or genes related to CHD (n = 251 genes) acquired from DisGeNET database. The data were analyzed by using the chi-square test. ***, *P*< 0.001 *vs.* genome group. **B** The proportions of m^6^A methylation target genes in neonatal rat hearts among all coding genes (genome, n = 21657 genes) or genes related to pulmonary stenosis (PS, n = 105 genes), atrioventricular septal defects (AVSD, n = 57 genes), ventricular septal defects (VSD, n = 419 genes), tetralogy of Fallot (TOF, n = 266 genes), atrial septal defects (ASD, n = 381 genes), and patent ductus arteriosus (PDA, n = 520 genes). The data were analyzed by using the chi-square test. ***, *P*< 0.001 *vs.* genome group. **C-D** Co-expression of *Mettl3* and cardiac development-related genes in mouse embryonic hearts from E9.5 to E16.5 was analyzed by using the latest mouse organogenesis spatiotemporal transcriptome atlas (MOSTA: https://db.cngb.org/stomics/mosta/). **C**, The heart region was annotated based on a spatially constrained-clustering algorithm and was specifically highlighted in red. **D**, Spatial visualization of the expressions of *Mettl3* and cardiac development-related genes in mouse embryo regions across E9.5 to E16.5, with the cardiac regions highlighted and other regions soft-masked. The color range indicates the expression level of individual genes. Dark blue indicates lower expression and white or orange indicates higher expression.

First, we performed bioinformatics analysis to explore the potential association of m^6^A RNA methylation with cardiovascular development and CHDs. We acquired m^6^A-methylated transcripts from rat neonatal hearts from the Gene Expression Omnibus database (GSE162545) and further mapped them to genes related to various CHDs from the DisGeNET database. Compared with the proportion of m^6^A methylation target genes in the genome (34.4%), m^6^A methylation target genes accounted for a higher proportion of CHD-related genes (49.0%) (Figure 1A), especially in genes related to pulmonary stenosis (60.0%), atrioventricular septal defects (56.1%), ventricular septal defects (53.0%), tetralogy of Fallot (48.5%), atrial septal defects (48.0%), and patent ductus arteriosus (46.5%) (Figure 1B). Considering that METTL3 is the core catalytic subunit of the m^6^A-methyltransferase complex, we next analyzed the expression of *Mettl3* and cardiac development-related genes in the heart region during mouse embryonic development using the data from the latest spatiotemporal transcriptome atlas (MOSTA: https://db.cngb.org/stomics/mosta/) 35. As a result, various critical cardiac development genes (*Gata5*, *Hand1*, *Tbx20*, *Ttn*, *etc*.) in embryonic hearts show at least a partial, correlated expression pattern with cardiac *Mettl3* expression during the embryonic heart development (Figure 1C-D). Such co-expression patterns of *Mettl3* and cardiac development genes suggested the potential correlation of *Mettl3*-mediated m^6^A RNA modification with the gene expression regulations related to cardiac development. Thus, METTL3-catalyzed m^6^A RNA methylation is likely correlated with cardiac development and CHDs, which we will further investigate in the following sections.

### Prenatal alcohol exposure downregulated METTL3-involved methyltransferase complex expression in mouse embryonic hearts

**Figure 2:**
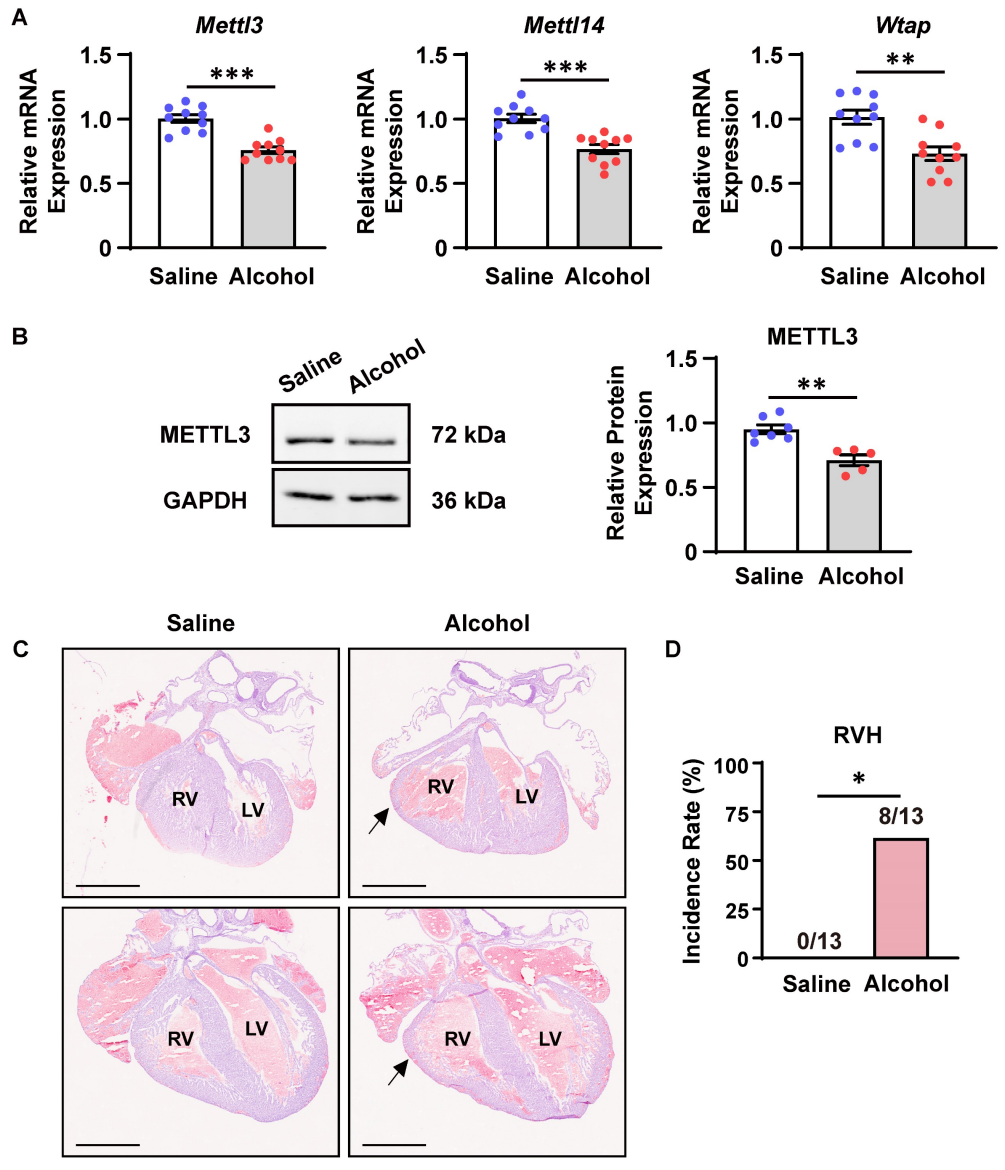
** Prenatal alcohol exposure downregulated METTL3-involved methyltransferase complex expression in mouse embryonic hearts. A** Quantitative analysis of mRNA expression in the hearts of mouse embryos exposed to alcohol or saline at E8.5-9.5 and collected at E10.5 was performed by RT‒qPCR. n = 10 per group, and each sample was obtained from 4 embryos. The data are presented as the mean ± SEM and were analyzed by using an unpaired two-tailed Student's *t* test. **, *P*< 0.01, ***, *P*< 0.001. **B** Representative Western blotting and quantitative analysis of METTL3 expression in the hearts of mouse embryos exposed to alcohol or saline at E8.5-9.5 and collected at E10.5. *n* = 7 *vs.* 5 per group, and each sample was obtained from 4 embryos. The data are presented as the mean ± SEM and were analyzed by using an unpaired two-tailed Student's *t* test. **, *P*< 0.01. **C** Representative hematoxylin-eosin staining images of hearts at E18.5 from mouse embryos exposed to alcohol or saline at E8.5-9.5. RV, right ventricle; LV, left ventricle. The black arrows indicate ventricular hypoplasia. Scale bar = 1 mm. **D** Quantitative analysis on the incidence of right ventricle hypoplasia (RVH) in mouse embryos exposed to alcohol or saline at E8.5-9.5. n =13 per group. The data were analyzed by using Fisher's exact test. *, *P*< 0.05.

Next, we explored whether CHD risk factors could modulate the m^6^A RNA methylation. As previously reported, maternal alcohol consumption increases the risk of CHDs in offspring 11, 36. The period from E8.5 to E10.5 marks a critical phase in heart development. Specifically, during E8.5 to E9.5, multi-source progenitor cells are recruited and the linear heart tube begins to undergo septation, leading to the formation of a four-chambered fetal heart. The development of the ventricular septum and outflow tract septum commences at E10.5 37, 38. Exposure to large amounts of alcohol during the critical period of cardiac morphogenesis (from E8.0 to E10.0) leads to cardiac malformations in a large proportion of offspring in mice 30. In accordance, we injected alcohol into pregnant females at E8.5-9.5 and detected the expression of major components of the m^6^A methyltransferase complex in offspring embryonic hearts at E10.5. As a consequence, prenatal alcohol exposure significantly downregulated *Mettl3*, *Mettl14*, and *Wtap* mRNA expression as well as METTL3 protein expression in mouse embryonic hearts (Figure 2A-B). Meanwhile, more than 60% of alcohol-exposed embryos at E18.5 indeed exhibited ventricular hypoplasia characterized by thin myocardium, especially in the right ventricles (Figure 2C-D), supporting the potential correlation of insufficient m^6^A RNA methylation with prenatal alcohol exposure-induced CHDs.

### Cardiovascular *Mettl3* deficiency results in postnatal lethality in mice

**Figure 3:**
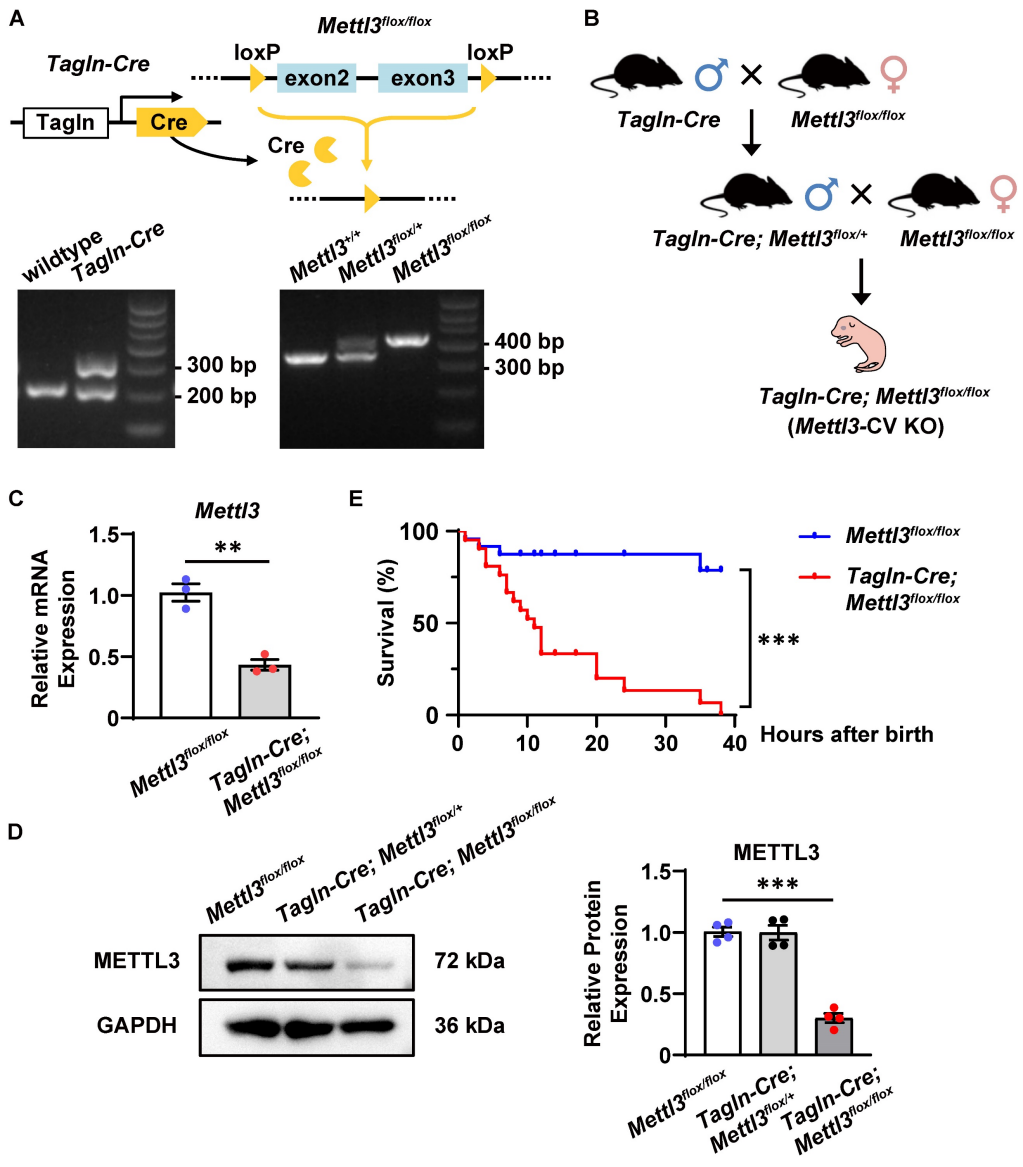
***Mettl3* deficiency driven by *Tagln-Cre* causes postnatal lethality in mice. A** Schematic representation of the strategy used to generate cardiovascular-specific *Mettl3* knockout mice and representative images of the genotyping procedure. **B** The breeding strategy for generating cardiovascular-specific *Mettl3* knockout mice (*Tagln-Cre*; *Mettl3^ flox/flox^*, named *Mettl3*-CV KO). **C** Quantitative analysis of *Mettl3* mRNA expression in the hearts of *Mettl3*-CV KO and control mouse embryos at E10.5-11.5 was determined by RT‒qPCR. n = 3 per group, and each sample was obtained from 5 embryos. The data are presented as the mean ± SEM and were analyzed by using an unpaired two-tailed Student's *t* test. **, *P*< 0.01. **D** Representative Western blotting and quantitative analysis of METTL3 expression in the hearts of *Mettl3*-CV KO and control mouse embryos at E10.5-11.5. *n* = 3 per group, and each sample was obtained from 5 embryos. The data are presented as the mean ± SEM and were analyzed by using an unpaired two-tailed Student's *t* test. ***, *P*< 0.001. **E** Kaplan‒Meier survival curves of *Mettl3*-CV KO (red line) and control mice (blue line) after birth. n =21 *vs.* 24*.* The data were analyzed by using the Gehan-Breslow-Wilcoxon test. ***, *P*< 0.001.

As previously reported, TAGLN (also named SM22α) is strongly expressed in the embryonic heart at the early stages of cardiac development (from E8.0 to E13.5) and is also expressed in vascular smooth muscle cells and endothelial cells differentiated from embryonic stem cells 39-41. Therefore, *Tagln-Cre* transgenic mice were utilized to efficiently intervene in cardiovascular gene expression 42-45. Accordingly, we generated cardiovascular-specific *Mettl3* knockout mice (*Tagln-Cre*; *Mettl3^flox/flox^*, named *Mettl3*-CV KO) through crossbreeding *Mettl3*-floxed mice with the *Tagln-Cre* transgenic line (Figure 3A-B). The conditional knockout was validated by measuring METTL3 expression in cardiac tissues from mouse embryos at E10.5-11.5. As expected, METTL3 expression was significantly downregulated in the hearts of *Mettl3*-CV KO mice compared with those of *Mettl3^flox/flox^* controls (Figure 3C-D). Interestingly, the number of *Mettl3*-CV KO embryos before delivery corresponded to the expected ratio for Mendelian inheritance (Table 1). However, compared with those of control littermates, more than 70% of the neonatal *Mettl3*-CV KO mice died within 12 hours after birth, and no mice survived after 40 hours (Figure 3E). Consequently, the deletion of cardiovascular* Mettl3* led to postnatal lethality in mice.

### Cardiovascular *Mettl3* deficiency causes congenital cardiac defects

**Figure 4:**
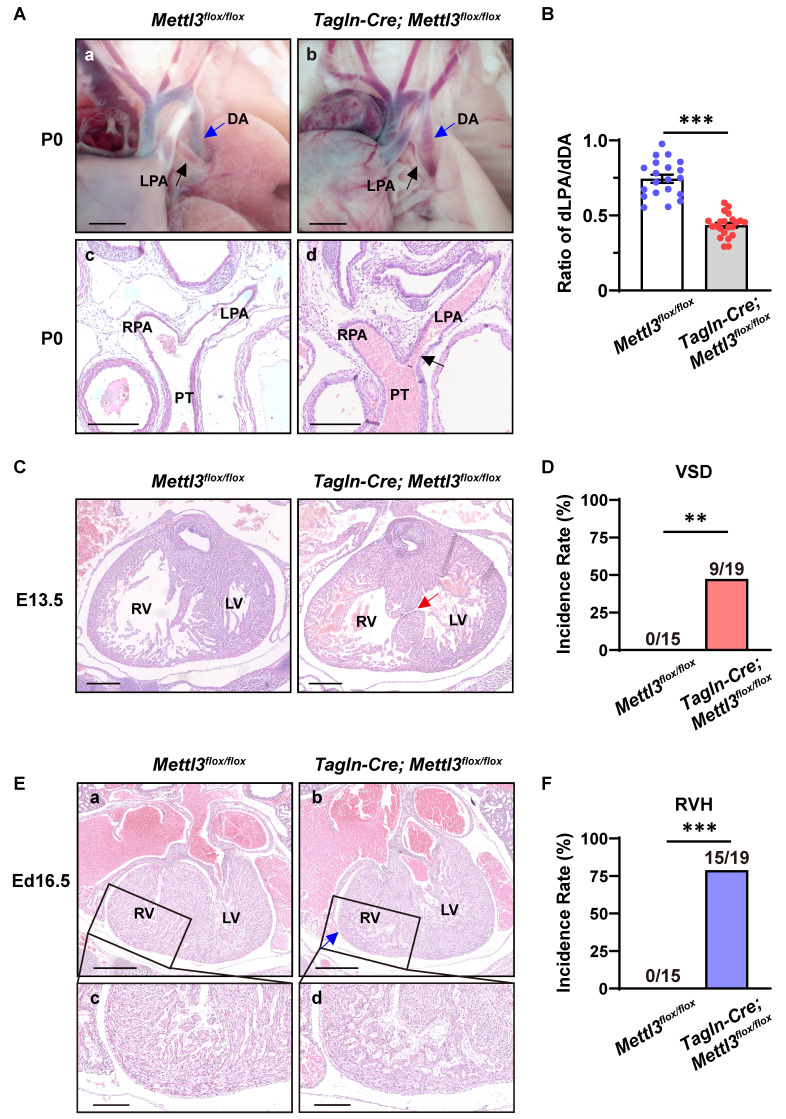
** Cardiovascular-specific *Mettl3* knockout mice exhibit congenital cardiac defects. A a-b**, Representative images of the left pulmonary artery of *Mettl3*-CV KO and control mice at postnatal day (P) 0. Scale bar = 500 µm. LPA, left pulmonary artery, black arrow; DA, descending aorta, blue arrow. **c-d**, Representative hematoxylin-eosin staining images of the bifurcation of the pulmonary trunk in *Mettl3*-CV KO and control mice at P0. The black arrow indicates the narrowing of the LPA. Scale bar = 250 µm. LPA, left pulmonary artery; RPA, right pulmonary artery; PT, pulmonary trunk. **B** Quantitative analysis on the ratios of the diameter of the left pulmonary artery to the diameter of the corresponding descending aorta (dLPA/dDA) of *Mettl3*-CV KO and control mice at P0. n =20 *vs.* 22. The data are presented as the mean ± SEM and were analyzed using an unpaired two-tailed Student's *t* test. ***, *P*< 0.001. **C** Representative hematoxylin-eosin staining images of hearts from *Mettl3*-CV KO and control mouse embryos at E13.5. The red arrow indicates the ventricular septal defect. Scale bar = 250 µm. RV, right ventricle; LV, left ventricle. **D** Quantitative analysis on the incidence of ventricular septal defects (VSD) in *Mettl3*-CV KO and control mice. n =15 *vs.* 19. The data were analyzed by using Fisher's exact test. **, *P*< 0.01. **E a-b**, Representative hematoxylin-eosin staining images of hearts from *Mettl3*-CV KO and control mouse embryos at E16.5. The blue arrow indicates ventricular hypoplasia. Scale bar = 500 µm. RV, right ventricle; LV, left ventricle. **c-d**, Enlarged view of the right ventricular wall. Scale bar = 200 µm. **F** Quantitative analysis on the incidence of right ventricle hypoplasia (RVH) in *Mettl3*-CV KO and control mice. n =15 *vs.* 19. The data were analyzed by using Fisher's exact test. ***, *P*< 0.001.

To further explore the cause of postnatal death, we performed *ex vivo* dissection and histological analysis of cardiovascular tissues from neonatal mice and embryos. As a result, *Mettl3*-CV KO mice exhibited severe left pulmonary stenosis (Figure 4A). The ratio of the diameter of the left pulmonary artery to the diameter of the corresponding descending aorta (dLPA/dDA) is considerably applied to evaluate the development and function of the left pulmonary artery 28, 29. We found that dLPA/dDA was markedly lower in the* Mettl3*-CV KO mice than in their control littermates (control *vs. Mettl3*-CV KO = 0.745 ± 0.027 *vs.* 0.435 ± 0.016, *P*<0.001) (Figure 4B). A decrease in this ratio may indicate pulmonary artery dysplasia and abnormal hemodynamics, which are harmful to normal cardiac function and the efficiency of oxygen delivery. In addition, approximately 47% of the *Mettl3*-CV KO mice exhibited ventricular septal defects (Figure 4C-D), while approximately 79% of the *Mettl3*-CV KO mice exhibited right ventricle hypoplasia concomitant with a thin ventricular wall, suggesting noncompaction of the ventricular myocardium (Figure 4E-F). Taken together, these congenital cardiac defects, which are suggestive of anomalies in the right ventricle and outflow tract, might be the cause of postnatal death in *Mettl3*-CV KO mice.

Since cell proliferation and apoptosis substantially correlate with cardiac development 46, we accordingly analyzed the expression of genes related to proliferation and apoptosis in the hearts and outflow tracts of *Mettl3*-CV KO and control mouse embryos at E10.5-11.5. As a consequence, the expression of PCNA and Cyclin D1 were both downregulated, suggesting the suppression of cell proliferation, in the hearts and outflow tracts of *Mettl3*-CV KO compared with control tissues (Figure S1A-B). In addition, *Mettl3* knockout also decreased the mRNA levels of apoptosis inhibitor *Bcl2* (Figure S1A). Thus, *Mettl3* deficiency inhibits cell proliferation but promotes cell apoptosis, which might be associated with cardiac defects in *Mettl3*-CV KO mice.

### METTL3 catalyzes m^6^A RNA methylation of the transcripts of *Sox4*, *Sox11* and *Mef2a*

**Figure 5:**
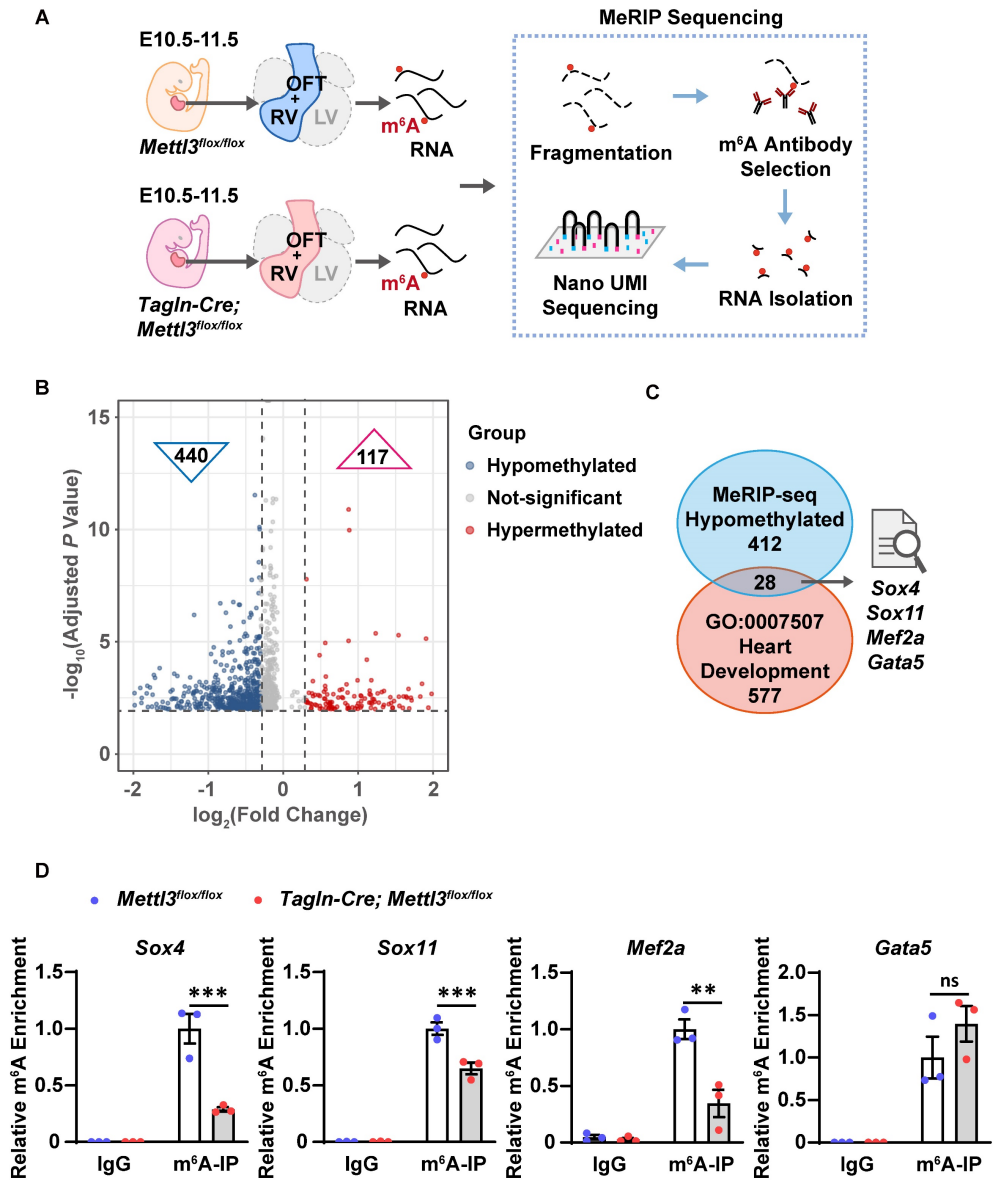
***Mettl3* deficiency causes hypomethylation of the *Sox4*, *Sox11*, and *Mef2a* transcripts in mouse embryonic hearts. A** Schematic diagram of the MeRIP-seq analysis used to identify m^6^A methylation target genes in mouse embryonic hearts. **B** Volcano plot showing genes corresponding to the differentially m^6^A-methylated transcripts detected by MeRIP-seq analysis in the hearts of *Mettl3*-CV KO and control mouse embryos at E10.5-11.5. The abscissa axis is log_2_ (fold change), and the ordinate axis is [-log_10_ (adjusted *P* value)]. The gray dots indicate the genes without differentially methylated m^6^A peaks, the blue dots indicate the genes with differentially hypomethylated m^6^A peaks in the *Mettl3*-CV KO group, and the red dots indicate the genes with differentially hypermethylated m^6^A peaks in the *Mettl3*-CV KO group. **C** Schematic diagram of screening m^6^A methylation target genes involved in congenital cardiac defects. **D** MeRIP-qPCR analysis of m^6^A enrichment in the transcripts of *Mef2a*, *Sox4*,* Sox11*, and *Gata5* in the hearts of *Mettl3*-CV KO and control mouse embryos at E10.5-11.5. *n* = 3 per group, and each sample was obtained from 15 embryos. The data are presented as the mean ± SEM and were analyzed by using two-way ANOVA followed by Tukey's test for post hoc comparisons. **, *P*< 0.01, ***, *P*< 0.001. ns = not significant.

To identify additional m^6^A methylation target genes involved in congenital cardiac defects in *Mettl3*-CV KO mice, we isolated right ventricle and outflow tract tissues, the main parts of the observed cardiac defects, from E10.5-11.5 *Mettl3*-CV KO and control embryos to perform unbiased m^6^A-specific methylated RNA immunoprecipitation (MeRIP) combined with high-throughput sequencing analysis (Figure 5A). As a result, 440 genes with differentially hypomethylated m^6^A peaks and 117 genes with differentially hypermethylated m^6^A peaks were identified (Figure 5B and Dataset S1-2). Gene Ontology analyses were performed (Figure S2A-B). Through mapping with genes related to "heart development" in Gene Ontology functional categories, we further enriched 28 candidates from hypomethylated genes (Dataset S3). We searched for reports on knockout mice of the 28 hypomethylated cardiovascular-specific candidates and summarized the major cardiovascular phenotypes and lethality of gene knockout (Dataset S4). Among them, *Gata5*,* Mef2a*, *Sox4* or *Sox11* deficiency have been shown to cause similar phenotypes with *Mettl3*-CV KO mice (Figure 5C). *Gata5* deficiency resulted in thinning ventricular wall in mouse embryos, while *Mef2a* deficiency resulted in right ventricular dilation, causing postnatal lethality 47, 48. *Sox4* and *Sox11* deficiency leads to ventricular septal defects and outflow tract malformations, resulting in embryonic or perinatal death 49, 50.

Thus, we validated the m^6^A enrichment of *Gata5*,* Mef2a*, *Sox4* and *Sox11* mRNAs in the embryonic hearts of *Mettl3*-CV KO mice and control littermates at E10.5-11.5 by using MeRIP-qPCR and found that *Mettl3* deficiency significantly reduced m^6^A levels in the transcripts of *Mef2a*,* Sox4* and *Sox11* but not in those of *Gata5* (Figure 5D). *Mef2a*,* Sox4*, and *Sox11* are targets of METTL3 catalytic m^6^A RNA methylation and could mediate METTL3-involved regulation of embryonic heart development.

### METTL3-catalyzed m^6^A RNA methylation facilitates the protein expression of SOX4, SOX11, and MEF2A

**Figure 6:**
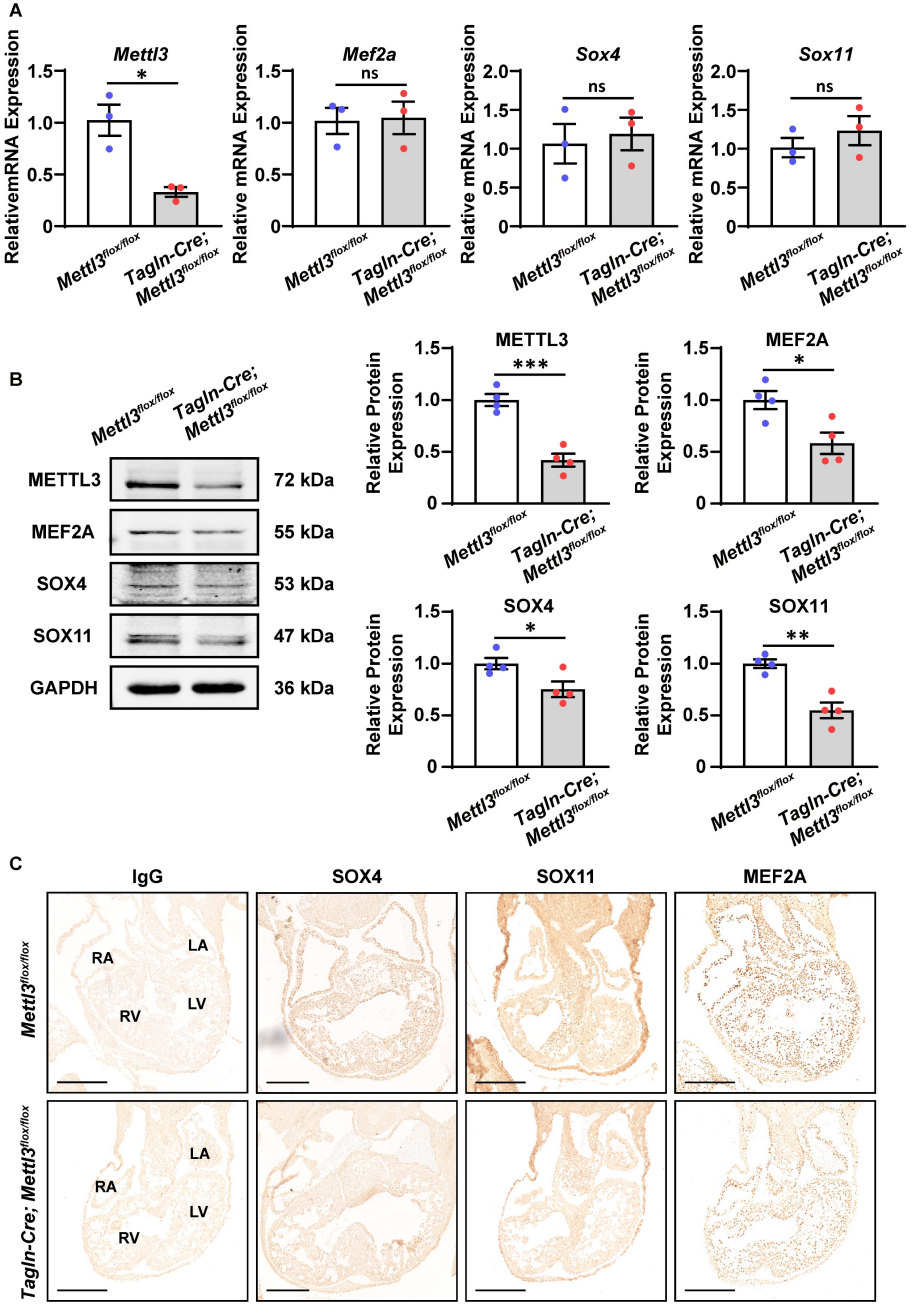
***Mettl3* deficiency downregulates the expression of SOX4, SOX11, and MEF2A proteins in mouse embryonic hearts. A** Quantitative analysis of mRNA expression in the hearts of *Mettl3*-CV KO and control mouse embryos at E10.5-11.5 was performed by RT‒qPCR. n = 3 per group, and each sample was obtained from 5 embryos. The data are presented as the mean ± SEM and were analyzed by using an unpaired two-tailed Student's *t* test. *, *P*< 0.05. ns = not significant. **B** Representative Western blotting and quantitative analysis of protein expression in the hearts of *Mettl3*-CV KO and control mouse embryos at E10.5-11.5. *n* = 4 per group, and each sample was obtained from 5 embryos. The data are presented as the mean ± SEM and were analyzed by using an unpaired two-tailed Student's *t* test. *, *P*< 0.05, **, *P*< 0.01, ***, *P*< 0.001. **C** Immunohistochemical staining images of SOX4, SOX11, and MEF2A in the hearts of *Mettl3*-CV KO and control mouse embryos at E10.5. Scale bar = 250 µm.

Next, we explored how m^6^A RNA methylation affects the expression of these target genes. Real-time PCR revealed that the mRNA levels of *Mef2a*, *Sox4*, and *Sox11* were not affected by *Mettl3* deficiency in the right ventricle and outflow tract tissues at E10.5-11.5 (Figure 6A and Figure S3A). Meanwhile, Western blotting analysis revealed that the protein expression of MEF2A, SOX4, and SOX11 was markedly downregulated in *Mettl3*-CV KO hearts compared with control tissues, while GATA5 expression was not affected (Figure 6B and Figure S3B). These results indicated that METTL3-catalyzed m^6^A RNA methylation facilitated the expression of these target genes at the protein level rather than at the mRNA level, possibly due to the m^6^A-enhanced translation pathway. Moreover, immunohistochemical staining also confirmed that the robust expression of the MEF2A, SOX4, and SOX11 proteins in the outflow tract and the atrial/ventricular myocardium was decreased by *Mettl3* deficiency (Figure 6C). *Mettl3* deficiency-induced downregulation of MEF2A, SOX4, and SOX11 expression could lead to congenital cardiac defects, including left pulmonary stenosis, ventricular septal defects, and right ventricular hypoplasia.

## Discussion

**Figure 7:**
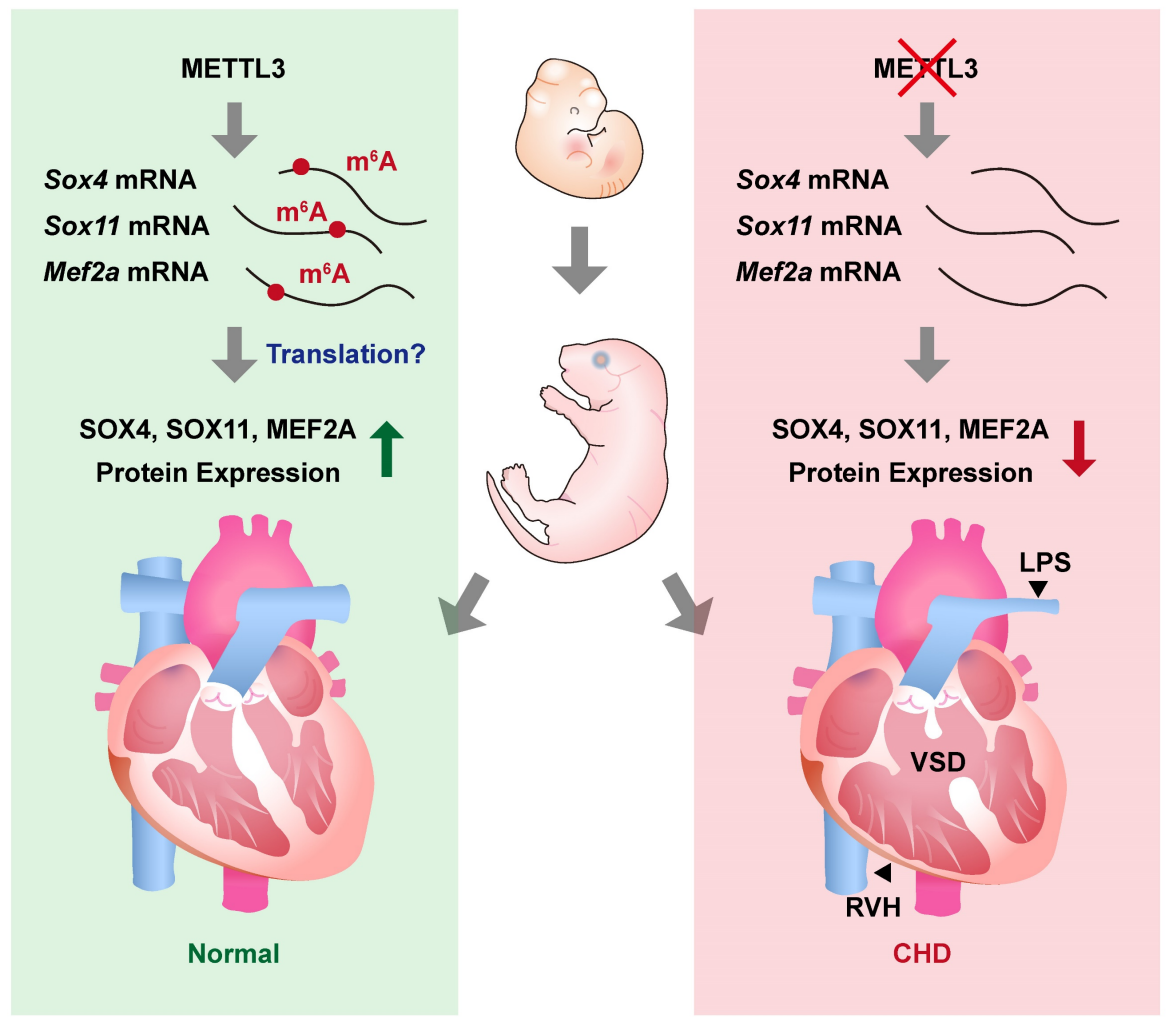
** METTL3 and m^6^A RNA modification are necessary for embryonic cardiovascular development.** METTL3 catalyzes the formation of m^6^A RNA methylation on transcripts of *Sox11*, *Sox4*, and *Mef2a* and ensures the normal embryonic development of cardiovascular systems. On the other hand, cardiovascular-specific METTL3 deficiency causes downregulation of SOX4, SOX11, and MEF2A, and leads to congenital cardiac defects including left pulmonary stenosis (LPS), ventricular septal defects (VSD) and right ventricular hypoplasia (RVH) in mice.

In this study, we revealed the essential role of METTL3-catalyzed m^6^A RNA methylation in cardiovascular development. We found that cardiovascular-specific *Mettl3* knockout mice died postnatally and exhibited congenital cardiac defects in the right ventricle and outflow tract, including left pulmonary stenosis, ventricular septal defects, and right ventricular hypoplasia (Figure 7). Moreover, the transcripts of *Sox4*, *Sox11*, and *Mef2a*, the critical transcription factors involved in cardiac development, were identified as the targets of METTL3-catalyzed m^6^A RNA methylation, whereas the congenital cardiac defects in cardiovascular-specific *Mettl3* knockout mice could be attributed to *Mettl3* deficiency-induced downregulation of SOX4, SOX11 and MEF2A expression.

The major contribution of the present study was the discovery of the role of epigenetic modifications on RNA in cardiac development and the occurrence of CHDs. Only approximately one-third of all CHD cases are attributed to aberrant gene mutation 11, whereas extra-genomic factors governing gene expression play a predominant role in the pathogenesis of CHDs alternatively. Emerging evidence has demonstrated that epigenetic modifications are associated with cardiac development and CHDs. For example, DNMT3B-mediated DNA hypermethylation, HDAC3-induced histone deacetylation, and BRG1-mediated chromatin remodeling can all significantly downregulate the expression of the transcription factor GATA4 and subsequently cause syndromic and nonsyndromic congenital cardiac defects, such as tetralogy of Fallot and ventricular septal defects 12, 51, 52. In addition to these reported epigenetic modifications, we utilized *Tagln-Cre*-driven cardiovascular-specific *Mettl3* knockout mice and found that METTL3-catalyzed m^6^A RNA methylation, the most common epigenetic modification on RNA, was substantially involved in cardiac development and that METTL3 deficiency directly resulted in congenital cardiac defects in the right ventricle and outflow tract, including left pulmonary stenosis, ventricular septal defects and right ventricular hypoplasia. The thinning ventricle and defective ventricular septum impaired the pumping function of the heart, while the narrowed pulmonary artery increased pulmonary circulation resistance. All of these defects exacerbated the cardiovascular dysfunction in newborn *Mettl3*-CV KO mice and consequently led to postnatal death 53, 54. Meanwhile, through the macroscopic morphological analysis of the cardiovascular system in newborn mice, we have excluded other possible causes of mortality, including patent ductus arteriosus, aortic arch stenosis, and other cardiovascular abnormalities. Of interest, previous studies focusing on cardiomyocytes have shown that *β-MHC-Cre*-induced METTL3 deficiency hardly impairs cardiac development, but prevents mature cardiomyocyte hypertrophy 55. Given the expression peak of *β-MHC* (E16) occurs later than that of *Tagln* (E10.5) in heart region 40, 56, the existence of METTL3 in cardiomyocytes during the initial stage (E8.5-10.5) of embryonic cardiac morphogenesis in *β-MHC-Cre*-induced METTL3 deficient embryos might be an important reason for the discrepancy in the phenotypes between *β-MHC-Cre; Mettl3^flox/flox^* mice and *Tagln-Cre; Mettl3^flox/flox^* mice. In addition, the expression of *β-MHC* is restricted to cardiomyocytes, while *Tagln* is expressed in multiple cell types including cardiomyocytes and vascular smooth muscle cells 39, 57. Thus, we could not exclude the potential contribution of METTL3 deficiency in VSMCs to the cardiac malformations in *Tagln-Cre; Mettl3^flox/flox^* mice, which requires further investigation.

The transcripts of *Sox4*, *Sox11*, and *Mef2a* have been identified as targets of m^6^A RNA methylation. Notably, ventricular septal defects and pulmonary stenosis are ascribed to malformations of the outflow tract 46. Previous studies have suggested that *Sox4* and *Sox11* are involved in outflow tract development. Deficiencies in *Sox4* and *Sox11* inhibit the migration of neural crest cells, as well as the epithelial-mesenchymal transition, and thereby lead to outflow tract malformations in mice, such as ventricular septum defects and pulmonary trunk stenosis 49, 50. Moreover, structural and functional defects in cardiac sarcomeres serve as major causes of ventricular hypoplasia 58. *Mef2a* has also been reported to play a pivotal role in the morphogenesis of cardiac sarcomeres, whereas *Mef2a* deficiency significantly causes postnatal lethality due to right ventricular hypoplasia and chamber dilation 47. Here, we found that reduced METTL3-catalyzed m^6^A RNA methylation downregulated *Sox4*, *Sox11*, and *Mef2a* expression. Thus, we propose that the congenital cardiac defects in cardiovascular-specific *Mettl3* knockout mice may be caused by insufficient expression of *Sox4*, *Sox11*, and *Mef2a*. However, the screening of candidate genes was restricted by our strategy, which was based on previous reports. Whether other hypomethylated candidates are also involved in cardiac malformations in *Mettl3*-CV KO embryos requires further investigation. Of note, we also identified 117 genes with differentially hypermethylated m^6^A peaks in the hearts of *Mettl3*-CV KO mouse embryos at E10.5-11.5 through MeRIP-seq analysis. Although, regrettably, GO analysis revealed no significant enrichment of hypermethylated transcripts in biological processes related to heart development, whether these hypermethylated genes mediated some other effects of *Mettl3* deficiency is still uncertain.

In addition, we found left ventricular hypoplasia existing in some of the *Mettl3*-CV KO mice as well, but less significantly than the incidence of right ventricular dysplasia. Since there is a lack of statistical significance in left ventricular hypoplasia at the current stage, whether *Mettl3* deficiency significantly caused left ventricular hypoplasia required further investigation dependent on the increased sample size. *Mef2a* also participates in left ventricular morphogenesis 59, 60, thus METTL3-regulated MEF2A expression might also mediate left ventricular development, but still requires further exploration.

The m^6^A-modified transcripts can modulate corresponding gene expression by altering mRNA stability and splicing as well as affecting the transcription and translation processes 18, 20. In the present study, we excluded the influence of m^6^A modification on the mRNA stability and transcription of *Sox4*, *Sox11*, and *Mef2a,* as evidenced by the lack of changes in the expression of these genes at the mRNA level. However, substantial evidence has indicated that SOX4, SOX11, and MEF2A protein expression is dependent on METTL3-catalyzed m^6^A RNA methylation, therefore, we hypothesized that m^6^A modification of these gene transcripts facilitates translation. In addition, a recent study reported that METTL3-catalyzed m^6^A RNA modification is important for maintaining the integrity of heterochromatin in mouse embryonic stem cells, thereby revealing a mechanism of heterochromatin regulation in embryonic development 61. The alteration of heterochromatin is related to chromatin remodeling, an epigenetic modification related to cardiac development and CHDs. Thus, the congenital cardiac defects we observed in cardiovascular-specific *Mettl3* knockout mice might also be related to heterochromatin changes that further regulate gene expression, although further investigation is needed for verification.

The incidence of CHDs is associated with various environmental factors or personal behaviors, such as air pollution, chemicals, radiation, viral infection, dietary intake, smoking, and alcohol consumption, which might directly modulate epigenetic modifications, particularly m^6^A RNA methylation 62-64. We found that prenatal alcohol exposure caused the downregulation of METTL3-involved methyltransferase complex in mouse embryonic hearts and remarkably improved the incidence of ventricular hypoplasia, partially in line with the phenotypes in *Mettl3*-CV KO embryos, indicating that the downregulation of METTL3 would be the potential causal factor to alcohol-induced cardiac malformation. Unfortunately, we did not observe ventricular septal defect in 13 embryos from two maternal mice with alcohol exposure, probably due to the limited sample size. In addition to our results, PM2.5 and environmental chemical (e.g., bisphenol A) exposure also downregulates METTL3 expression and decreases global m^6^A methylation 63, 65. These findings suggest that maternal exposure to these environmental toxicants during pregnancy might reduce fetal METTL3-m^6^A RNA methylation and increase the risk of CHDs. METTL3 expression is also modulated by methionine and S-adenosylmethionine, whereas dietary methionine restriction markedly downregulates METTL3 expression 66. Guaranteeing methionine intake during gestation might be beneficial for fetal cardiac development and preventing the occurrence of CHDs. Thus, environmental and behavioral intervention to maintain the essential levels of fetal METTL3 expression or m^6^A RNA methylation might be a potential strategy to prevent CHDs, especially during pregnancy.

## Supplementary Material

Supplementary figures, tables and datasets.

## Figures and Tables

**Table 1:** Genotypic distribution of survival offsprings generated by crossing *Tagln-Cre; Mettl3^flox/+^* mice with *Mettl3^flox/flox^* mice

Age	*Tagln-Cre; Mettl3^flox/flox^*	*Tagln-Cre; Mettl3^flox/+^*	*Mettl3^flox/flox^*	*Mettl3^flox/+^*	Total
E10.5-11.5	49 (27%)	48 (27%)	42 (23%)	42 (23%)	181
E12.5-18.5	53 (25%)	58 (27%)	54 (25%)	49 (23%)	214
P1	2 (2%) ***	32 (31%)	35 (34%)	34 (33%)	103

E, embryonic day; P, postnatal day. Data were analyzed by using the chi-square test. ***, *P*< 0.001 *vs*. other genotypes.

## Abbreviations

ALKBH5: alkB homolog 5
BCL2: B-cell lymphoma-2
FTO: fat mass and obesity-associated protein
CHD: congenital heart disease
GATA5: GATA binding protein 5
m^6^A: N^6^-methyladenosine
MEF2A: myocyte enhancer factor 2A
MeRIP: m^6^A-specific methylated RNA-immunoprecipitation
METTL3/14: methyltransferase-like 3/14
PCNA: proliferating cell nuclear antigen
SOX4/11: SRY-box transcription factor 4/11
WTAP: Wilms' tumor 1-associating protein
