# Supplementary material for: Cardiovascular Mettl3 Deficiency Causes Congenital Cardiac Defects and Postnatal Lethality in Mice
Source: Int J Biol Sci. 2025 Mar 10;21(6):2430–45. doi: 10.7150/ijbs.100941 (PMC12035893; doi:10.7150/ijbs.100941)
Supplement: Supplementary file 1 — Supplementary figures, tables and datasets. [file ijbsv21p2430s1.pdf]

Supplementary Material

Cardiovascular Mettl3 Deficiency Causes Congenital Cardiac Defects and Postnatal Lethality in Mice

Qianqian Feng, Lihua Qi, Jiaqi Huang, Zhigang Dong, Fang Yu, Jing Zhang, Jun Zhan, Hongquan Zhang, Wengong Wang, Yong Zhou, Zhongzhou Yang, Yuan Zhou, Wei Kong, Yi Fu

This PDF includes:

Supplementary Figure 1-3

Supplementary Table 1-2

Supplementary Dataset 1-4

Supplementary Figures and Figure Legends

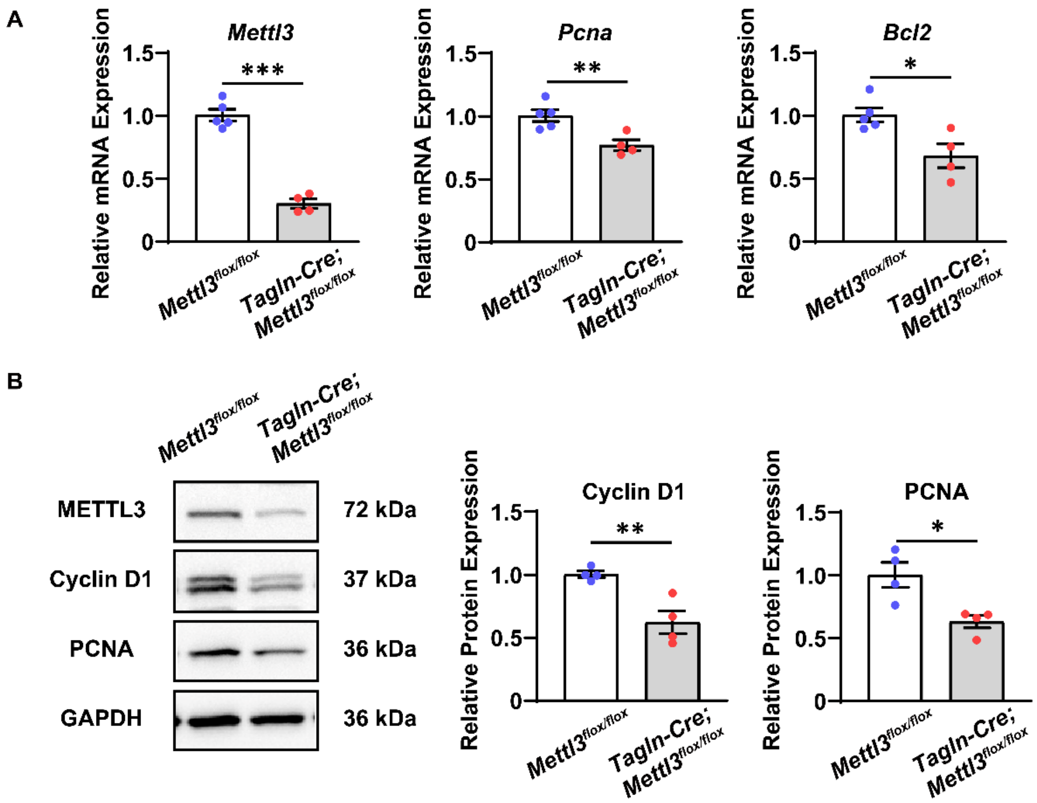

Figure S1. Changes in genes related to proliferation and apoptosis in the hearts and outflow tracts of mouse embryos caused by *Mettl3* deficiency.

A Quantitative analysis of mRNA expression in the hearts and outflow tracts of *Mettl3*-CV KO and control mouse embryos at E10.5-11.5 was performed by RT–qPCR. n = 5 *vs.* 4, and each sample was obtained from 3 embryos. The data are presented as the mean ± SEM and were analyzed by using an unpaired two-tailed Student's *t* test. *, P< 0.05, **, P< 0.01, ***, P< 0.001.

B Representative Western blotting and quantitative analysis of protein expression in the hearts and outflow tracts of *Mettl3*-CV KO and control mouse embryos at E10.5-11.5. n = 4 per group, and each sample was obtained from 5 embryos. The data are presented as the mean ± SEM and were analyzed by using an unpaired two-tailed Student's *t* test. *, P< 0.05, **, P< 0.01.

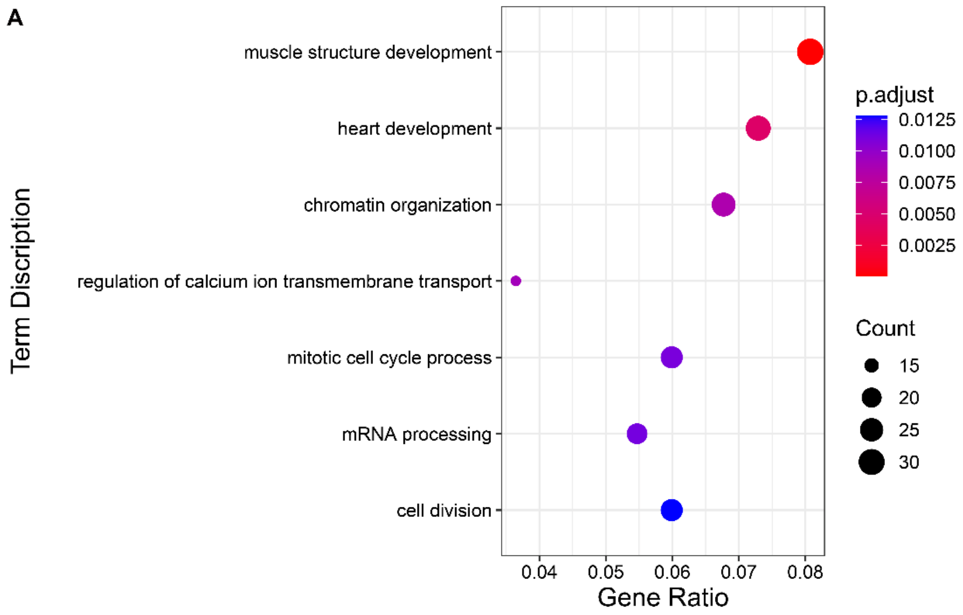

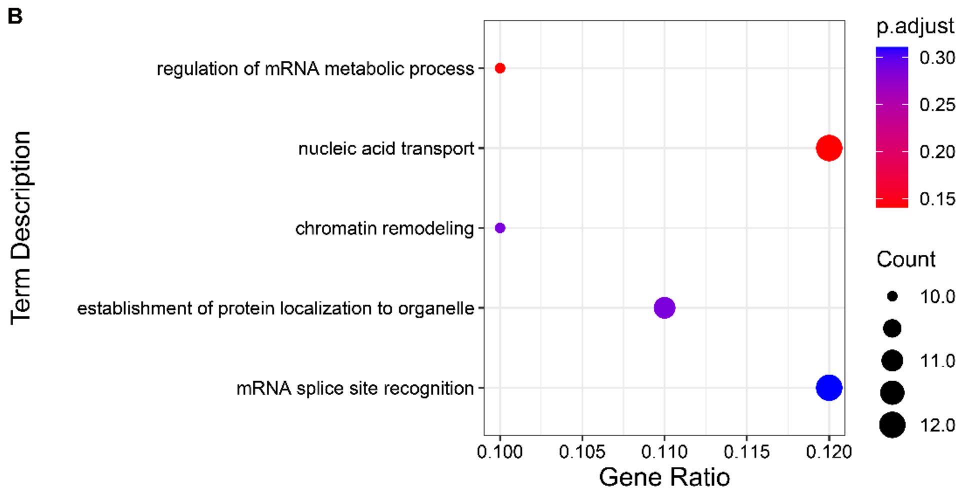

Figure S2. Gene Ontology analysis of genes with differentially methylated m^{6}A peaks in the hearts of *Mettl3*-CV KO embryos.

A Gene Ontology analysis of genes with differentially hypomethylated m^{6}A peaks detected by MeRIP-seq analysis in the hearts of *Mettl3-CV* KO mouse embryos at E10.5-11.5.

B Gene Ontology analysis of genes with differentially hypermethylated m^{6}A peaks detected by MeRIP-seq analysis in the hearts of *Mettl3-CV* KO mouse embryos at E10.5-11.5.

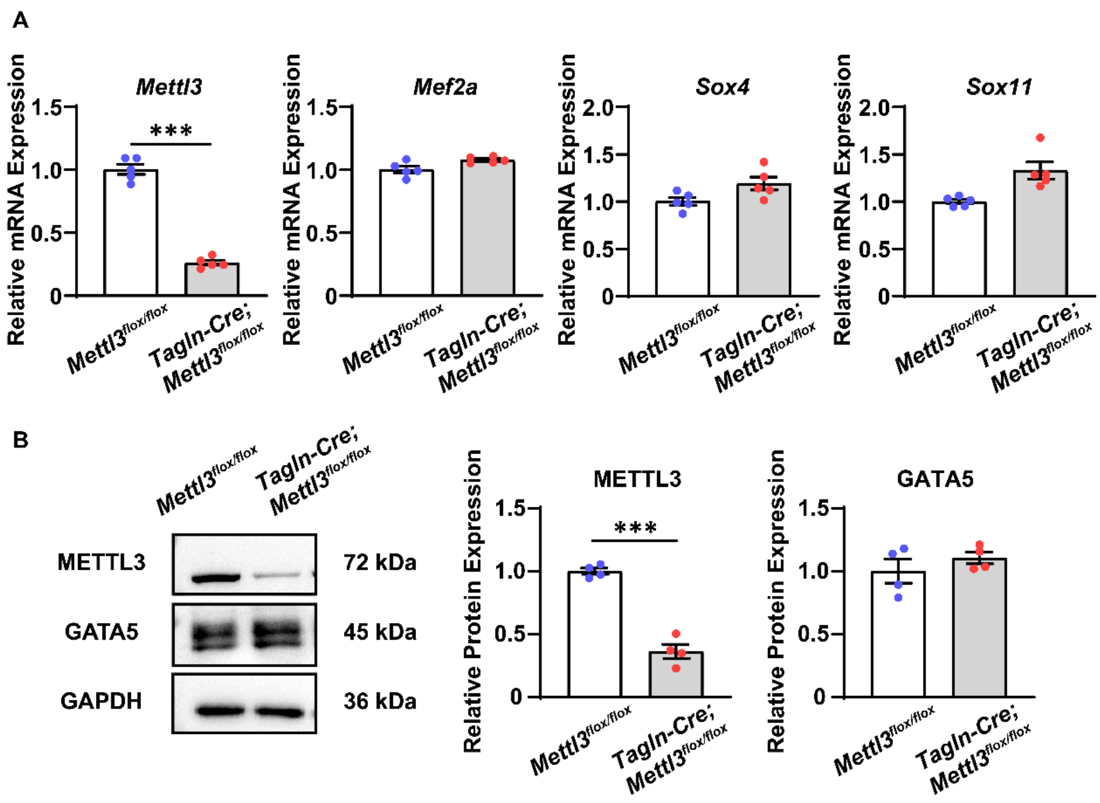

Figure S3. Changes in gene expression in mouse embryonic hearts caused by *Mettl3* deficiency.

A Quantitative analysis of mRNA expression in the right ventricle and outflow tract tissues of *Mettl3*-CV KO and control mouse embryos at E10.5-11.5 was performed by RT–qPCR. n = 5 per group, and each sample was obtained from 3 embryos. The data are presented as the mean ± SEM and were analyzed by using an unpaired two-tailed Student's *t* test. ***, $P< 0.001$.

B Representative Western blotting and quantitative analysis of protein expression in the hearts of *Mettl3*-CV KO and control mouse embryos at E10.5-11.5. *n* = 4 per group, and each sample was obtained from 5 embryos. The data are presented as the mean ± SEM and were analyzed by using an unpaired two-tailed Student's *t* test. ***, $P< 0.001$.

Supplementary Tables

Table S1. Primers used for genotyping (5’→3’)

Tagln-Cre	
P1	ATTTGCCTGCATTACCGGTCG
P2	CAGCATTGCTGTCACTTGGTC
P3	CAAATGTTGCTTGTCTGGTG
P4	GTCAGTCGAGTGCACAGTTT
Mettl3 $^{\mathbf{flox/flox}}$	
flox 3’Forward	GCACTCAATAGAGAGAGGATCTGGAAG
flox 3’Reverse	CTCTGGTTATCGTCATCGAAGACCAAAC

Table S2. Primers used for qPCR

Target	Forward primer (5'→3')	Reverse primer (5'→3')
Gapdh	AGGTCGGTGTGAACGGATTTG	TGTAGACCATGTAGTTGAGGTCA
Mettl3	GGACTCTGGGCACTTGGATTT	ATCAGTGGGCAAGGTCAAGG
Mettl14	CTGAGAGTGCGGATAGCATTG	GAGCAGATGTATCATAGGAAGCC
Wtap	TAGACCCAGCGATCAACTTGT	CCTGTTTGGCTATCAGGCGTA
Mef2a	AGTAGCGGAGACTCGGAATTG	ATGCATCGTACACAGCTCCT
Mef2a (MeRIP)	TGGAGTGAAGCATTCTGAAGGT	TTTCCCTGGATCACACTCGG
Sox4	ACGCCTTTATGGTGTGGTCG	CCGACTTCACCTTCTTTCGC
Sox4 (MeRIP)	GCTGGCGAAATTTTCTGTGC	GCTGGCGAAATTTTCTGTGC
Sox11	ACAGCGAGAAGATCCCGTTC	CCGTCTTGGGCTTTTTGCG
Sox11 (MeRIP)	TGCAGTGTTAATGCTCAGGG	ACATGTGGAGATTGATCACACGA
Gata5 (MeRIP)	GCTGGCGAAATTTTCTGTGC	GCTGGCGAAATTTTCTGTGC
Bcl2	GTCGCTACCGTCGTGACTTC	CAGACATGCACCTACCCAGC
Pcna	TTTGAGGCACGCCTGATCC	GGAGACGTGAGACGAGTCCAT

Supplementary Dataset

Dataset S1. List of genes with differentially hypermethylated m^{6}A peaks in the hearts of *Mettl3*-CV KO mouse embryos.

Gene ID	Name
ENSMUSG00000018796	acyl-CoA synthetase long-chain family member 1 (Acsl1)
ENSMUSG00000020994	Pinin (Pnn)
ENSMUSG00000026987	bromodomain adjacent to zinc finger domain, 2B (Baz2b)
ENSMUSG00000031010	ubiquitin specific peptidase 9, X chromosome (Usp9x)
ENSMUSG00000026207	SPEG complex locus (Speg)
ENSMUSG00000039197	adenosine kinase (Adk)
ENSMUSG00000098812	microRNA 7578 (Mir7578)
ENSMUSG00000051339	RIKEN cDNA 2900026A02 gene (2900026A02Rik)
ENSMUSG00000031871	cadherin 5 (Cdh5)
ENSMUSG00000033365	importin 13 (Ipo13)
ENSMUSG00000020464	polyribonucleotide nucleotidyltransferase 1 (Pnpt1)
ENSMUSG00000021546	heterogeneous nuclear ribonucleoprotein K (Hnrnpk)
ENSMUSG00000037058	polyadenylate-binding protein-interacting protein 2 (Paip2)
ENSMUSG00000042719	N(alpha)-acetyltransferase 25, NatB auxiliary subunit (Naa25)
ENSMUSG00000014195	DnaJ heat shock protein family (Hsp40) member C7 (Dnajc7)
ENSMUSG00000022214	DDB1 and CUL4 associated factor 11 (Dcaf11)
ENSMUSG00000014426	mitogen-activated protein kinase kinase kinase 4 (Map3k4)
ENSMUSG00000028626	collagen, type IX, alpha 2 (Col9a2)
ENSMUSG00000000078	Kruppel-like transcription factor 6 (Klf6)
ENSMUSG00000052798	nucleoporin 107 (Nup107)
ENSMUSG00000031446	cullin 4A (Cul4a)
ENSMUSG00000026926	peptidase (mitochondrial processing) alpha (Pmpca)
ENSMUSG00000026918	bromodomain containing 3 (Brd3)
ENSMUSG00000072612	predicted gene 10382 (Gm10382)
ENSMUSG00000045868	GTPase, very large interferon inducible 1 (Gvin1)
ENSMUSG00000031715	SWI/SNF related, matrix associated, actin dependent regulator of chromatin, subfamily a, member 5 (Smarca5)
ENSMUSG00000028483	small nuclear RNA activating complex, polypeptide 3 (Snapc3)
ENSMUSG00000079402	predicted gene 3020 (Gm3020)
ENSMUSG00000018765	FMR1 autosomal homolog 2 (Fxr2)
ENSMUSG00000021706	zinc finger, FYVE domain containing 16 (Zfyve16)
ENSMUSG00000030201	low density lipoprotein receptor-related protein 6 (Lrp6)
ENSMUSG00000092167	predicted gene 3696 (Gm3696)
ENSMUSG00000020307	cell division cycle 34 (Cdc34)
ENSMUSG00000029686	cullin 1 (Cul1)
ENSMUSG00000026596	ABL proto-oncogene 2, non-receptor tyrosine kinase (Abl2)
ENSMUSG00000032575	mesencephalic astrocyte-derived neurotrophic factor (Manf)
ENSMUSG00000032527	propionyl Coenzyme A carboxylase, beta polypeptide (Pccb)

Gene ID	Name
ENSMUSG00000069833	AHNAK nucleoprotein (Ahnak)
ENSMUSG00000026153	family with sequence similarity 135, member A (Fam135a)
ENSMUSG00000027523	GNAS complex locus (Gnas)
ENSMUSG00000028127	ATP-binding cassette, sub-family D member 3 (Abcd3)
ENSMUSG00000059248	septin 9 (Septin9)
ENSMUSG00000021196	phosphofructokinase, platelet (Pfkp)
ENSMUSG00000032212	SAFB-like, transcription modulator (Sltm)
ENSMUSG00000055884	Fanconi anemia, complementation group M (Fancm)
ENSMUSG00000031393	methyl CpG binding protein 2 (Mecp2)
ENSMUSG00000030035	WW domain binding protein 1 (Wbp1)
ENSMUSG00000028484	PC4 and SFRS1 interacting protein 1 (Psip1)
ENSMUSG00000042167	terminal nucleotidyltransferase 2 (Tent2)
ENSMUSG00000042901	axin interactor, dorsalization associated (Aida)
ENSMUSG00000047721	bolA family member 2 (Bola2)
ENSMUSG00000055044	PDZ and LIM domain 1 (elfin) (Pdlim1)
ENSMUSG00000020160	Meis homeobox 1 (Meis1)
ENSMUSG00000038784	CCR4-NOT transcription complex, subunit 4 (Cnot4)
ENSMUSG00000033499	La ribonucleoprotein 4B (Larp4b)
ENSMUSG00000005836	GATA binding protein 6 (Gata6)
ENSMUSG00000075254	heart development protein with EGF-like domains 1 (Heg1)
ENSMUSG00000027287	synaptosomal-associated protein 23 (Snap23)
ENSMUSG00000001855	nucleoporin 214 (Nup214)
ENSMUSG00000004347	phosphodiesterase 1C (Pde1c)
ENSMUSG00000059586	NSE2/MMS21 homolog, SMC5-SMC6 complex SUMO ligase (Nsmce2)
ENSMUSG00000046743	FAT atypical cadherin 4 (Fat4)
ENSMUSG00000034998	forkhead box N2 (Foxn2)
ENSMUSG00000029246	phosphoribosyl pyrophosphate amidotransferase (Ppat)
ENSMUSG00000020850	pre-mRNA processing factor 8 (Prpf8)
ENSMUSG00000021270	heat shock protein 90, alpha (cytosolic), class A member 1 (Hsp90aa1)
ENSMUSG00000021597	SMC5-SMC6 complex localization factor 1 (Slf1)
ENSMUSG00000040940	Rho guanine nucleotide exchange factor 1 (Arhgef1)
ENSMUSG00000118671	epiplakin 1 (Eppk1)
ENSMUSG00000078676	exon junction complex subunit (Casc3)
ENSMUSG00000039987	putative homeodomain transcription factor 2 (Phtf2)
ENSMUSG00000020741	clustered mitochondria homolog (Cluh)
ENSMUSG00000033031	cell proliferation regulating inhibitor of protein phosphatase 2A (Cip2a)
ENSMUSG00000071226	CECR2, histone acetyl-lysine reader (Cecr2)
ENSMUSG00000020074	cell division cycle and apoptosis regulator 1 (Ccar1)
ENSMUSG00000028034	far upstream element (FUSE) binding protein 1 (Fubp1)

Gene ID	Name
ENSMUSG00000051675	tripartite motif-containing 32 (Trim32)
ENSMUSG00000008348	ubiquitin C (Ubc)
ENSMUSG00000028559	oxysterol binding protein-like 9 (Osbpl9)
ENSMUSG00000035530	eukaryotic translation initiation factor 1 (Eif1)
ENSMUSG00000002265	paternally expressed 3 (Peg3)
ENSMUSG00000018102	H2B clustered histone 4 (H2bc4)
ENSMUSG00000053414	hormonally upregulated Neu-associated kinase (Hunk)
ENSMUSG00000043535	Senataxin (Setx)
ENSMUSG00000050565	torsin A interacting protein 2 (Tor1aip2)
ENSMUSG00000018651	transcriptional adaptor 2A (Tada2a)
ENSMUSG00000022263	triple functional domain (PTPRF interacting) (Trio)
ENSMUSG00000024754	cell migration inducing hyaluronidase 2 (Cemip2)
ENSMUSG00000033623	polycomb group ring finger 3 (Pcgf3)
ENSMUSG00000016386	metallophosphoesterase domain containing 2 (Mpped2)
ENSMUSG00000075028	PR domain containing 11 (Prdm11)
ENSMUSG00000035851	YTH domain containing 1 (Ythdc1)
ENSMUSG00000021313	ryanodine receptor 2, cardiac (Ryr2)
ENSMUSG00000051390	zinc finger and BTB domain containing 22 (Zbtb22)
ENSMUSG00000028068	IQ motif containing GTPase activating protein 3 (Iqgap3)
ENSMUSG00000036246	Gem-interacting protein (Gmip)
ENSMUSG00000032422	sorting nexin 14 (Snx14)
ENSMUSG00000026605	centromere protein F (Cenpf)
ENSMUSG00000092541	Predicted gene 20537 (Gm20537)
ENSMUSG00000003161	Sorcin (Sri)
ENSMUSG00000033799	transcription activation suppressor family member 2 (Tasor2)
ENSMUSG00000029153	OCIA domain containing 2 (Ociad2)
ENSMUSG00000037519	protein tyrosine phosphatase, receptor type, f polypeptide (PTPRF), interacting protein (liprin), alpha 1 (Ppfia1)
ENSMUSG00000066036	ubiquitin protein ligase E3 component n-recognin 4 (Ubr4)
ENSMUSG00000074564	
ENSMUSG00000094293	
ENSMUSG00000096736	
ENSMUSG00000085166	
ENSMUSG00000095891	
ENSMUSG00000105985	
ENSMUSG00000095547	
ENSMUSG00000113836	
ENSMUSG00000095186	
ENSMUSG00000075015	
ENSMUSG00000108880	
ENSMUSG00000096385	
ENSMUSG00000091165	

Dataset S2. List of genes with differentially hypomethylated m^{6}A peaks in the hearts of *Mettl3*-CV KO mouse embryos.

Gene ID	Name
ENSMUSG00000034675	drebrin 1(Dbn1)
ENSMUSG00000057637	PR domain containing 2, with ZNF domain (Prdm2)
ENSMUSG00000051331	calcium channel, voltage-dependent, L type, alpha 1C subunit (Cacna1c)
ENSMUSG00000029050	ski sarcoma viral oncogene homolog (avian) (Ski)
ENSMUSG00000056758	high mobility group AT-hook 2 (Hmga2)
ENSMUSG00000033060	LIM domain only 7 (Lmo7)
ENSMUSG00000026199	ankyrin repeat and zinc finger domain containing 1 (Ankzf1)
ENSMUSG00000021243	FCF1 rRNA processing protein (Fcf1)
ENSMUSG00000039361	phosphatidylinositol binding clathrin assembly protein (Picalm)
ENSMUSG00000000555	integrin alpha 5 (fibronectin receptor alpha) (Itga5)
ENSMUSG00000049047	armadillo repeat containing, X-linked 3 (Armcx3)
ENSMUSG00000021870	sarcolemma associated protein (Slmap)
ENSMUSG00000038538	ubinuclein 2 (Ubn2)
ENSMUSG00000026208	Desmin (Des)
ENSMUSG00000017677	WD repeat and SOCS box-containing 1 (Wsb1)
ENSMUSG00000005442	capicua transcriptional repressor (Cic)
ENSMUSG00000003166	DiGeorge syndrome critical region gene 2 (Dgcr2)
ENSMUSG00000021770	sterile alpha motif domain containing 8 (Samd8)
ENSMUSG00000035863	paralemmin (Palm)
ENSMUSG00000042644	inositol 1,4,5-triphosphate receptor 3 (Itpr3)
ENSMUSG00000028745	capping actin protein of muscle Z-line subunit beta (Capzb)
ENSMUSG00000054640	solute carrier family 8 (sodium/calcium exchanger), member 1 (Slc8a1)
ENSMUSG00000025137	phosphate cytidylyltransferase 2, ethanolamine (Pcyt2)
ENSMUSG00000024063	limb-bud and heart (Lbh)
ENSMUSG00000038212	major facilitator superfamily domain containing 14B (Mfsd14b)
ENSMUSG00000028614	NDC1 transmembrane nucleoporin (Ndc1)
ENSMUSG00000021938	paraspeckle protein 1 (Pspc1)
ENSMUSG00000024213	nudix hydrolase 3 (Nudt3)
ENSMUSG00000045103	dystrophin, muscular dystrophy (Dmd)
ENSMUSG00000037815	catenin alpha 1 (Ctnna1)
ENSMUSG00000020849	tyrosine 3-monooxygenase/tryptophan 5-monooxygenase activation protein, epsilon polypeptide (Ywhae)
ENSMUSG00000006932	catenin beta 1 (Ctnnb1)
ENSMUSG00000034101	catenin delta 1 (Ctnnd1)
ENSMUSG00000019977	Hbs1-like (S. cerevisiae) (Hbs1l)
ENSMUSG00000047963	starch binding domain 1 (Stbd1)

Gene ID	Name
ENSMUSG00000028977	castor zinc finger 1 (Casz1)
ENSMUSG00000049550	CAP-GLY domain containing linker protein 1 (Clip1)
ENSMUSG00000028576	intraflagellar transport 74 (Ift74)
ENSMUSG00000048874	PHD finger protein 3 (Phf3)
ENSMUSG00000042650	alkB homolog 5, RNA demethylase (Alkbh5)
ENSMUSG00000015627	GATA binding protein 5 (Gata5)
ENSMUSG00000030189	Y box protein 3 (Ybx3)
ENSMUSG00000009575	chromobox 5 (Cbx5)
ENSMUSG00000038462	ubiquinol-cytochrome c reductase, Rieske iron-sulfur polypeptide 1 (Uqcrfs1)
ENSMUSG00000027715	cyclin A2 (Ccna2)
ENSMUSG00000015143	actinin, alpha 1 (Actn1)
ENSMUSG00000039087	ras responsive element binding protein 1 (Rreb1)
ENSMUSG00000020523	family with sequence similarity 114, member A2 (Fam114a2)
ENSMUSG00000029190	DNA segment, Chr 5, ERATO Doi 579, expressed (D5Ertd579e)
ENSMUSG00000034007	S phase cyclin A-associated protein in the ER (Scaper)
ENSMUSG00000092470	Predicted gene 20518 (Gm20518)
ENSMUSG00000039376	synaptopodin 2-like (Synpo2l)
ENSMUSG00000035047	KRI1 homolog (Kri1)
ENSMUSG00000101645	catenin delta 1 (Ctnnd1)
ENSMUSG00000042354	guanine nucleotide binding protein nucleolar 3 (Gnl3)
ENSMUSG00000062960	kinase insert domain protein receptor (Kdr)
ENSMUSG00000028649	microtubule-actin crosslinking factor 1 (Macf1)
ENSMUSG00000038774	activating signal cointegrator 1 complex subunit 3 (Ascc3)
ENSMUSG00000063015	cyclin I (Ccni)
ENSMUSG00000021375	kinesin family member 13A (Kif13a)
ENSMUSG00000038763	alpha-kinase 3 (Alpk3)
ENSMUSG00000030204	DEAD box helicase 47 (Ddx47)
ENSMUSG00000025134	Aly/REF export factor (Alyref)
ENSMUSG00000022961	Son DNA binding protein (Son)
ENSMUSG00000021693	kinesin family member 2A (Kif2a)
ENSMUSG00000030079	RuvB-like AAA ATPase 1 (Ruvbl1)
ENSMUSG00000063632	SRY (sex determining region Y)-box 11 (Sox11)
ENSMUSG00000044308	ubiquitin protein ligase E3 component n-recognin 3 (Ubr3)
ENSMUSG00000020668	kinesin family member 3C (Kif3c)
ENSMUSG00000051817	SRY (sex determining region Y)-box 12 (Sox12)
ENSMUSG00000032911	chondroitin sulfate proteoglycan 4 (Cspg4)
ENSMUSG00000022800	forty-two-three domain containing 1 (Fyttd1)
ENSMUSG00000032349	ELOVL fatty acid elongase 5 (Elovl5)
ENSMUSG00000118491	predicted readthrough transcript (NMD candidate), 44505 (Gm44505)

Gene ID	Name
ENSMUSG00000094475	predicted gene 14430 (Gm14430)
ENSMUSG00000076431	SRY (sex determining region Y)-box 4 (Sox4)
ENSMUSG00000055485	microtubule crosslinking factor 2 (Mtcl2)
ENSMUSG00000022263	triple functional domain (PTPRF interacting) (Trio)
ENSMUSG00000001280	trans-acting transcription factor 1 (Sp1)
ENSMUSG00000026131	dystonin (Dst)
ENSMUSG00000079139	predicted gene 4204 (Gm4204)
ENSMUSG00000021288	kinesin light chain 1 (Klc1)
ENSMUSG00000026478	laminin, gamma 1 (Lamc1)
ENSMUSG00000111409	ring finger protein 26 (Rnf26)
ENSMUSG00000005087	CD44 antigen(Cd44)
ENSMUSG00000026353	UBX domain protein 4 (Ubxn4)
ENSMUSG00000029191	replication factor C (activator 1) 1 (Rfc1)
ENSMUSG00000041638	GCN1 activator of EIF2AK4 (Gcn1)
ENSMUSG00000027777	schwannomin interacting protein 1 (Schip1)
ENSMUSG00000044496	RIKEN cDNA 2510039O18 gene (2510039O18Rik)
ENSMUSG00000022897	dual-specificity tyrosine phosphorylation regulated kinase 1a (Dyrk1a)
ENSMUSG00000022893	ADAM metallopeptidase with thrombospondin type 1 motif 1 (Adamts1)
ENSMUSG00000118770	predicted gene, 22450 (Gm22450)
ENSMUSG00000016477	E2F transcription factor 3 (E2f3)
ENSMUSG00000094638	predicted gene 21972 (Gm21972)
ENSMUSG00000119278	predicted gene, 23092 (Gm23092)
ENSMUSG00000054199	gon-4 like (Gon4l)
ENSMUSG00000022994	adenylate cyclase 6 (Adcy6)
ENSMUSG00000020780	signal recognition particle 68 (Srp68)
ENSMUSG00000032279	isocitrate dehydrogenase 3 (NAD^+) alpha (Idh3a)
ENSMUSG00000026564	serine/threonine/tyrosine interacting like 2 (Styxl2)
ENSMUSG00000119556	predicted gene, 23523 (Gm23523)
ENSMUSG00000028098	ring finger protein 115 (Rnf115)
ENSMUSG00000026987	bromodomain adjacent to zinc finger domain, 2B (Baz2b)
ENSMUSG00000040433	zinc finger and BTB domain containing 38 (Zbtb38)
ENSMUSG00000050953	gap junction protein, alpha 1 (Gja1)
ENSMUSG00000038156	spondin 1, (f-spondin) extracellular matrix protein (Spon1)
ENSMUSG00000025220	O-GlcNAcase (Oga)
ENSMUSG00000033788	Dysferlin (Dysf)
ENSMUSG00000034163	zinc finger, C3H1-type containing (Zfc3h1)
ENSMUSG00000036894	RAP2B, member of RAS oncogene family (Rap2b)
ENSMUSG00000056962	jumonji domain containing 6 (Jmjd6)
ENSMUSG00000053128	ring finger protein 26 (Rnf26)
ENSMUSG00000057110	Centriolin (Cntrl)

Gene ID	Name
ENSMUSG00000052915	male specific lethal 1 (Msl1)
ENSMUSG00000032998	forkhead box J3 (Foxj3)
ENSMUSG00000022010	TSC22 domain family, member 1 (Tsc22d1)
ENSMUSG00000039239	transforming growth factor, beta 2 (Tgfb2)
ENSMUSG00000036371	serpine1 mRNA binding protein 1 (Serbp1)
ENSMUSG00000031948	lysyl-tRNA synthetase 1 (Kars1)
ENSMUSG00000037820	transglutaminase 2, C polypeptide (Tgm2)
ENSMUSG00000053931	calponin 3, acidic (Cnn3)
ENSMUSG00000069793	schlafen 9 (Slfn9)
ENSMUSG00000028433	ubiquitin-associated protein 2 (Ubap2)
ENSMUSG00000024081	CCAAT/enhancer binding protein zeta (Cebpz)
ENSMUSG00000017707	serine incorporator 3 (Serinc3)
ENSMUSG00000079402	predicted gene 3020 (Gm3020)
ENSMUSG00000029405	G3BP stress granule assembly factor 2 (G3bp2)
ENSMUSG00000032352	leucine rich repeat containing 1 (Lrrc1)
ENSMUSG00000056076	eukaryotic translation initiation factor 3, subunit B (Eif3b)
ENSMUSG00000034462	polycystin 2, transient receptor potential cation channel (Pkd2)
ENSMUSG00000037266	arginine/serine rich protein 1 (Rsrp1)
ENSMUSG00000069539	SCY1-like 2 (S. cerevisiae) (Scyl2)
ENSMUSG00000040659	EF hand domain containing 2 (Efhd2)
ENSMUSG00000095195	predicted gene 3005 (Gm3005)
ENSMUSG00000062353	predicted gene 15772 (Gm15772)
ENSMUSG00000052428	transmembrane and coiled-coil domains 1 (Tmco1)
ENSMUSG00000020069	heterogeneous nuclear ribonucleoprotein H3 (Hnrnph3)
ENSMUSG00000032562	G protein subunit alpha i2 (Gnai2)
ENSMUSG00000024695	zinc finger protein 91 (Zfp91)
ENSMUSG00000061410	zinc finger, CCHC domain containing 14 (Zcchc14)
ENSMUSG00000027254	microtubule-associated protein 1 A (Map1a)
ENSMUSG00000027806	TSC22 domain family, member 2 (Tsc22d2)
ENSMUSG00000029064	guanine nucleotide binding protein (G protein), beta 1 (Gnb1)
ENSMUSG00000040097	FLYWCH-type zinc finger 1 (Flywch1)
ENSMUSG00000093674	ribosomal protein L41 (Rpl41)
ENSMUSG00000027425	lysine acetyltransferase 14 (Kat14)
ENSMUSG00000034681	RNA binding protein with serine rich domain 1 (Rnps1)
ENSMUSG00000016933	phospholipase C, gamma 1 (Plcg1)
ENSMUSG00000020542	myocardin (Myocd)
ENSMUSG00000093315	microRNA 5114 (Mir5114)
ENSMUSG00000031154	OTU domain containing 5 (Otud5)
ENSMUSG00000026851	cDNA sequence BC005624 (BC005624)
ENSMUSG00000038663	fibronectin type III and SPRY domain containing 2 (Fsd2)
ENSMUSG00000018428	A kinase anchor protein 1 (Akap1)
ENSMUSG00000022565	Plectin (Plec)

Gene ID	Name
ENSMUSG00000025364	proliferation-associated 2G4 (Pa2g4)
ENSMUSG00000001729	thymoma viral proto-oncogene 1 (Akt1)
ENSMUSG00000045598	zinc finger protein 553 (Zfp553)
ENSMUSG00000062202	BTB domain containing 9 (Btbd9)
ENSMUSG00000063870	chromodomain helicase DNA binding protein 4 (Chd4)
ENSMUSG00000078903	predicted gene 14391 (Gm14391)
ENSMUSG00000024298	zinc finger protein 871 (Zfp871)
ENSMUSG00000022110	succinate-Coenzyme A ligase, ADP-forming, beta subunit (Sucla2)
ENSMUSG00000022698	N(alpha)-acetyltransferase 50, NatE catalytic subunit (Naa50)
ENSMUSG00000051413	pleiomorphic adenoma gene-like 2 (Plagl2)
ENSMUSG00000044792	iron-sulfur cluster assembly 1 (Isca1)
ENSMUSG00000029587	zinc finger protein 12 (Zfp12)
ENSMUSG00000038371	SET binding factor 2 (Sbf2)
ENSMUSG00000113258	predicted gene 7446 (Gm7446)
ENSMUSG00000063200	nucleolar protein 7 (Nol7)
ENSMUSG00000022951	regulator of calcineurin 1 (Rcan1)
ENSMUSG00000021891	methyltransferase 6, methylcytidine (Mettl6)
ENSMUSG00000038733	WD repeat domain 26 (Wdr26)
ENSMUSG00000026988	WD repeat, SAM and U-box domain containing 1 (Wdsub1)
ENSMUSG00000021027	Ral GTPase activating protein, alpha subunit 1 (Ralgapa1)
ENSMUSG00000070544	topoisomerase (DNA) I (Top1)
ENSMUSG00000016382	plastin 3 (T-isoform) (Pls3)
ENSMUSG00000027722	AFG2 AAA ATPase homolog A (Afg2a)
ENSMUSG00000063754	predicted pseudogene 10136 (Gm10136)
ENSMUSG00000016319	solute carrier family 25 (mitochondrial carrier, adenine nucleotide translocator), member 5 (Slc25a5)
ENSMUSG00000074136	RIKEN cDNA 4930513N10 gene (4930513N10Rik)
ENSMUSG00000038116	PHD finger protein 20 (Phf20)
ENSMUSG00000038762	ATP-binding cassette, sub-family F member 1 (Abcf1)
ENSMUSG00000040325	DDB1 and CUL4 associated factor 1 (Dcaf1)
ENSMUSG00000041560	NOP53 ribosome biogenesis factor (Nop53)
ENSMUSG00000025034	tripartite motif-containing 8 (Trim8)
ENSMUSG00000090841	myosin, light polypeptide 6, alkali, smooth muscle and non-muscle (Myl6)
ENSMUSG00000056394	ligase I, DNA, ATP-dependent (Lig1)
ENSMUSG00000020697	ligase III, DNA, ATP-dependent (Lig3)
ENSMUSG00000050107	histone H3 associated protein kinase (Haspin)
ENSMUSG00000032803	carnitine deficiency-associated gene expressed in ventricle 3 (Cdv3)
ENSMUSG00000037519	protein tyrosine phosphatase, receptor type, f polypeptide (PTPRF), interacting protein (liprin), alpha 1 (Ppfia1)

Gene ID	Name
ENSMUSG00000055319	Sec23 interacting protein (Sec23ip)
ENSMUSG00000004665	calponin 2 (Cnn2)
ENSMUSG00000033577	myosin VI (Myo6)
ENSMUSG00000033228	SR-related CTD-associated factor 11 (Scaf11)
ENSMUSG00000073079	signal recognition particle 54A (Srp54a)
ENSMUSG00000039988	ankyrin repeat domain 13c (Ankrd13c)
ENSMUSG00000005893	nuclear receptor subfamily 2, group C, member 2 (Nr2c2)
ENSMUSG00000003119	cyclin dependent kinase 12 (Cdk12)
ENSMUSG00000062075	lamin B2 (Lmnb2)
ENSMUSG00000024687	oxysterol binding protein (Osbp)
ENSMUSG00000026207	SPEG complex locus (Speg)
ENSMUSG00000064037	GPN-loop GTPase 1 (Gpn1)
ENSMUSG00000017386	TNF receptor associated factor 4 (Traf4)
ENSMUSG00000039671	zinc finger, MYND-type containing 8 (Zmynd8)
ENSMUSG00000020694	tousled-like kinase 2 (Arabidopsis) (Tlk2)
ENSMUSG00000063556	predicted gene 10132 (Gm10132)
ENSMUSG00000034333	zinc finger, BED type containing 4 (Zbed4)
ENSMUSG00000037111	SET domain containing (lysine methyltransferase) 7 (Setd7)
ENSMUSG00000002820	autophagy related 4D, cysteine peptidase (Atg4d)
ENSMUSG00000059119	nucleosome assembly protein 1-like 4 (Nap1l4)
ENSMUSG00000051777	IQ motif containing J (Iqcj)
ENSMUSG00000095366	predicted gene 15247 (Gm15247)
ENSMUSG00000094090	predicted gene 8494 (Gm8494)
ENSMUSG00000025812	par-3 family cell polarity regulator (Pard3)
ENSMUSG00000027206	COP9 signalosome subunit 2 (Cops2)
ENSMUSG00000038486	synaptic vesicle glycoprotein 2a (Sv2a)
ENSMUSG00000032475	non-catalytic region of tyrosine kinase adaptor protein 1 (Nck1)
ENSMUSG00000005886	nuclear receptor coactivator 2 (Ncoa2)
ENSMUSG00000015568	lipoprotein lipase (Lpl)
ENSMUSG00000058594	F-box DNA helicase 1 (Fbh1)
ENSMUSG00000000631	myosin XVIIIA (Myo18a)
ENSMUSG00000112449	signal recognition particle 54B (Srp54b)
ENSMUSG00000024422	DEAH-box helicase 16 (Dhx16)
ENSMUSG00000038646	RNA guanine-7 methyltransferase activating subunit (Ramac)
ENSMUSG00000019362	DNA segment, Chr 8, ERATO Doi 738, expressed (D8Ertd738e)
ENSMUSG00000022803	popeye domain containing 2 (Popdc2)
ENSMUSG00000032232	cingulin-like 1 (Cgnl1)
ENSMUSG00000078552	doublecortin domain containing 2b (Dcdc2b)
ENSMUSG00000043909	transformation related protein 53 binding protein 1 (Trp53bp1)
ENSMUSG00000042502	CD2 cytoplasmic tail binding protein 2 (Cd2bp2)

Gene ID	Name
ENSMUSG00000018819	lymphocyte specific 1 (Lsp1)
ENSMUSG00000029622	actin related protein 2/3 complex, subunit 1B (Arpc1b)
ENSMUSG00000031448	ADP-ribosylhydrolase like 1 (Adprhl1)
ENSMUSG00000119516	predicted gene, 24464 (Gm24464)
ENSMUSG00000026991	plakophilin 4 (Pkp4)
ENSMUSG00000027522	syntaxin 16 (Stx16)
ENSMUSG00000048578	Malectin (Mlec)
ENSMUSG00000071532	predicted gene 10335 (Gm10335)
ENSMUSG00000026229	proteasome (prosome, macropain) 26S subunit, non-ATPase, 1 (Psmd1)
ENSMUSG00000038615	nuclear factor, erythroid derived 2,-like 1 (Nfe2l1)
ENSMUSG00000028033	potassium voltage-gated channel, subfamily Q, member 5 (Kcnq5)
ENSMUSG00000023089	NADH:ubiquinone oxidoreductase subunit A5 (Ndufa5)
ENSMUSG00000046185	zinc finger protein 84 (Zfp84)
ENSMUSG00000003099	protein phosphatase 5, catalytic subunit (Ppp5c)
ENSMUSG00000091537	translational machinery associated 7 (Tma7)
ENSMUSG00000051747	Titin (Ttn)
ENSMUSG00000024327	solute carrier family 39 (zinc transporter), member 7 (Slc39a7)
ENSMUSG00000038872	zinc finger homeobox 3 (Zfhx3)
ENSMUSG00000030850	arginyltransferase 1 (Ate1)
ENSMUSG00000038086	heat shock protein 2 (Hspb2)
ENSMUSG00000058672	tubulin, beta 2A class IIA (Tubb2a)
ENSMUSG00000042406	activating transcription factor 4 (Atf4)
ENSMUSG00000021733	solute carrier family 4, sodium bicarbonate cotransporter, member 7 (Slc4a7)
ENSMUSG00000046139	protein associated with topoisomerase II homolog 1 (yeast) (Patl1)
ENSMUSG00000035799	twist basic helix-loop-helix transcription factor 1 (Twist1)
ENSMUSG00000025261	HECT, UBA and WWE domain containing 1 (Huwe1)
ENSMUSG00000092607	sodium channel modifier 1 (Scnm1)
ENSMUSG00000015745	pleckstrin homology domain containing, family O member 1 (Plekho1)
ENSMUSG00000021613	hyaluronan and proteoglycan link protein 1 (Hapln1)
ENSMUSG00000033161	ATPase, Na^{+}/K^{+} transporting, alpha 1 polypeptide (Atp1a1)
ENSMUSG00000032060	crystallin, alpha B (Cryab)
ENSMUSG00000110131	predicted gene, 18066 (Gm18066)
ENSMUSG00000024079	eukaryotic translation initiation factor 2-alpha kinase 2 (Eif2ak2)
ENSMUSG00000078676	exon junction complex subunit (Casc3)
ENSMUSG00000075592	NYN domain and retroviral integrase containing (Nynrin)

Gene ID	Name
ENSMUSG00000031370	zinc finger (CCCH type), RNA binding motif and serine/arginine rich 2 (Zrsr2)
ENSMUSG00000036275	RIKEN cDNA 9530068E07 gene (9530068E07Rik)
ENSMUSG00000028034	far upstream element (FUSE) binding protein 1 (Fubp1)
ENSMUSG00000024077	striatin, calmodulin binding protein (Strn)
ENSMUSG00000050315	synaptopodin 2 (Synpo2)
ENSMUSG00000022191	drosha, ribonuclease type III (Drosha)
ENSMUSG00000079641	ribosomal protein L39 (Rpl39)
ENSMUSG00000063888	ribosomal protein L7-like 1 (Rpl7l1)
ENSMUSG00000026020	NOP58 ribonucleoprotein (Nop58)
ENSMUSG00000020522	microfibrillar-associated protein 3 (Mfap3)
ENSMUSG00000047649	RNA polymerase I subunit G (Polr1g)
ENSMUSG00000080076	H2A clustered histone 14, pseudogene (H2ac14-ps)
ENSMUSG00000063021	H2A clustered histone 15 (H2ac15)
ENSMUSG00000025326	ubiquitin protein ligase E3A (Ube3a)
ENSMUSG00000046707	casein kinase 2, alpha prime polypeptide (Csnk2a2)
ENSMUSG00000026260	NADH:ubiquinone oxidoreductase subunit A10 (Ndufa10)
ENSMUSG00000021614	versican (Vcan)
ENSMUSG00000096488	predicted gene 10409 (Gm10409)
ENSMUSG00000027272	ubiquitin protein ligase E3 component n-recognin 1 (Ubr1)
ENSMUSG00000033713	forkhead box N3 (Foxn3)
ENSMUSG00000043411	ubiquitin specific peptidase 48 (Usp48)
ENSMUSG00000017817	junctophilin 2 (Jph2)
ENSMUSG00000000568	heterogeneous nuclear ribonucleoprotein D (Hnrnpd)
ENSMUSG00000102422	Iqcj and Schip1 fusion protein (Iqschfp)
ENSMUSG00000029422	arginine/serine-rich coiled-coil 2 (Rsrc2)
ENSMUSG00000038170	phosphodiesterase 4D interacting protein (myomegalin) (Pde4dip)
ENSMUSG00000047921	trafficking protein particle complex 9 (Trappc9)
ENSMUSG00000027907	S100 calcium binding protein A11 (S100a11)
ENSMUSG00000068566	myeloid-associated differentiation marker (Myadm)
ENSMUSG00000016559	H3.3 histone B(H3f3b)
ENSMUSG00000063810	ALMS1, centrosome and basal body associated (Alms1)
ENSMUSG00000048376	coagulation factor II thrombin receptor (F2r)
ENSMUSG00000098387	PET117 homolog(Pet117)
ENSMUSG00000032612	ubiquitin specific peptidase 4 (proto-oncogene) (Usp4)
ENSMUSG00000037364	serrate RNA effector molecule homolog (Arabidopsis) (Srrt)
ENSMUSG00000046574	proline rich 12 (Prr12)
ENSMUSG00000025027	X-prolyl aminopeptidase (aminopeptidase P) 1, soluble (Xpnpep1)
ENSMUSG00000056121	fasciculation and elongation protein zeta 2 (Fez2)
ENSMUSG00000022889	mitochondrial ribosomal protein L39 (Mrpl39)

Gene ID	Name
ENSMUSG00000038648	cAMP responsive element binding protein 3-like 2 (Creb3l2)
ENSMUSG00000079481	NHS like 2 (Nhsl2)
ENSMUSG00000038773	KDM3B lysine (K)-specific demethylase 3B (Kdm3b)
ENSMUSG00000010914	pyruvate dehydrogenase complex, component X (Pdhx)
ENSMUSG00000058392	ribosomal RNA processing 1B (Rrp1b)
ENSMUSG00000018476	KDM1 lysine (K)-specific demethylase 6B (Kdm6b)
ENSMUSG00000018474	chromodomain helicase DNA binding protein 3 (Chd3)
ENSMUSG00000026895	NADH:ubiquinone oxidoreductase subunit A8 (Ndufa8)
ENSMUSG00000025203	stearoyl-Coenzyme A desaturase 2 (Scd2)
ENSMUSG00000117338	Small ribosomal subunit protein eS10 (Gm49804)
ENSMUSG00000061462	obscurin, cytoskeletal calmodulin and titin-interacting RhoGEF (Obscn)
ENSMUSG00000020706	FtsJ RNA 2'-O-methyltransferase 3 (Ftsj3)
ENSMUSG00000020902	netrin 1 (Ntn1)
ENSMUSG00000002948	mitogen-activated protein kinase kinase 7 (Map2k7)
ENSMUSG00000041408	WAPL cohesin release factor (Wapl)
ENSMUSG00000027620	RNA binding motif protein 39 (Rbm39)
ENSMUSG00000024513	methyl-CpG binding domain protein 2 (Mbd2)
ENSMUSG00000035478	methyl-CpG binding domain protein 3 (Mbd3)
ENSMUSG00000030403	vasodilator-stimulated phosphoprotein (Vasp)
ENSMUSG00000002870	minichromosome maintenance complex component 2 (Mcm2)
ENSMUSG00000041057	WD repeat domain 43 (Wdr43)
ENSMUSG00000024908	protein phosphatase 6, regulatory subunit 3 (Ppp6r3)
ENSMUSG00000033295	protein tyrosine phosphatase receptor type F (Ptprf)
ENSMUSG00000033871	peroxisome proliferative activated receptor, gamma, coactivator 1 beta (Ppargc1b)
ENSMUSG00000093930	3-hydroxy-3-methylglutaryl-Coenzyme A synthase 1 (Hmgcs1)
ENSMUSG00000033278	protein tyrosine phosphatase receptor type M (Ptprm)
ENSMUSG00000029729	zinc finger with KRAB and SCAN domains 1 (Zkscan1)
ENSMUSG00000008855	histone deacetylase 5 (Hdac5)
ENSMUSG00000001440	karyopherin subunit beta 1 (Kpnb1)
ENSMUSG00000034832	tet methylcytosine dioxygenase 3 (Tet3)
ENSMUSG00000037313	transforming, acidic coiled-coil containing protein 3 (Tacc3)
ENSMUSG00000022807	oxysterol binding protein-like 11 (Osbpl11)
ENSMUSG00000034974	death-associated protein kinase 3 (Dapk3)
ENSMUSG00000002833	HDGF like 2 (Hdgfl2)
ENSMUSG00000021175	cell division cycle associated 7 like (Cdca7l)
ENSMUSG00000032394	immunoglobulin superfamily, DCC subclass, member 3 (Igdcc3)
ENSMUSG00000036242	armadillo-like helical domain containing 4 (Armh4)
ENSMUSG00000069833	AHNAK nucleoprotein (Ahnak)

Gene ID	Name
ENSMUSG00000009112	BCL2 like 13 (Bcl2l13)
ENSMUSG00000048410	zinc finger protein 407 (Zfp407)
ENSMUSG00000021115	vaccinia related kinase 1 (Vrk1)
ENSMUSG00000066441	retinol dehydrogenase 11 (Rdh11)
ENSMUSG00000035295	WD repeat domain 38 (Wdr38)
ENSMUSG00000030557	myocyte enhancer factor 2A (Mef2a)
ENSMUSG00000026385	diazepam binding inhibitor (Dbi)
ENSMUSG00000022711	phosphomannomutase 2 (Pmm2)
ENSMUSG00000042043	tubulin cofactor A (Tbca)
ENSMUSG00000028780	sema domain, immunoglobulin domain (Ig), short basic domain, secreted, (semaphorin) 3C (Sema3c)
ENSMUSG00000026566	myelin protein zero-like 1 (Mpzl1)
ENSMUSG00000021796	bone morphogenetic protein receptor, type 1A (Bmpr1a)
ENSMUSG00000031865	dynactin 1 (Dctn1)
ENSMUSG00000064572	predicted gene, 25801 (Gm25801)
ENSMUSG00000026554	DDB1 and CUL4 associated factor 8 (Dcaf8)
ENSMUSG00000052698	talin 2 (Tln2)
ENSMUSG00000000787	DEAD box helicase 3, X-linked (Ddx3x)
ENSMUSG00000060679	mitochondrial ribosomal protein S9 (Mrps9)
ENSMUSG00000036968	canopy FGF signaling regulator 4 (Cnpy4)
ENSMUSG00000061436	homeodomain interacting protein kinase 2 (Hipk2)
ENSMUSG00000041852	transcription factor 20 (Tcf20)
ENSMUSG00000024238	zinc finger E-box binding homeobox 1 (Zeb1)
ENSMUSG00000050248	EvC ciliary complex subunit 2 (Evc2)
ENSMUSG00000002625	A kinase anchor protein 8-like (Akap8l)
ENSMUSG00000024073	baculoviral IAP repeat-containing 6 (Birc6)
ENSMUSG00000096992	predicted gene, 26788 (Gm26788)
ENSMUSG00000020640	intersectin 2 (Itsn2)
ENSMUSG00000022194	poly(A) binding protein, nuclear 1 (Pabpn1)
ENSMUSG00000018845	unc-45 myosin chaperone B (Unc45b)
ENSMUSG00000062997	ribosomal protein L35 (Rpl35)
ENSMUSG00000038943	protein regulator of cytokinesis 1 (Prc1)
ENSMUSG00000053414	hormonally upregulated Neu-associated kinase (Hunk)
ENSMUSG00000015994	farnesyltransferase, CAAX box, alpha (Fnta)
ENSMUSG00000040084	BUB1B, mitotic checkpoint serine/threonine kinase (Bub1b)
ENSMUSG00000039960	ras homolog family member U (Rhou)
ENSMUSG00000068036	afadin, adherens junction formation factor (Afdn)
ENSMUSG00000034574	dishevelled associated activator of morphogenesis 1 (Daam1)
ENSMUSG00000024754	cell migration inducing hyaluronidase 2 (Cemip2)
ENSMUSG00000011306	SURP and G patch domain containing 1 (Sugp1)
ENSMUSG00000033487	fibronectin type III domain containing 3A (Fndc3a)
ENSMUSG00000040479	diacylglycerol kinase zeta (Dgkz)

**Gene ID**	**Name**
ENSMUSG00000009207	lunapark, ER junction formation factor (Lnpk)
ENSMUSG00000039234	SEC24 homolog D, COPII coat complex component (Sec24d)
ENSMUSG00000086583	predicted pseudogene 15500 (Gm15500)
ENSMUSG00000035173	coiled-coil domain containing 186 (Ccdc186)
ENSMUSG00000028718	Scl/Tal1 interrupting locus (Stil)
ENSMUSG00000031790	matrix metallopeptidase 15 (Mmp15)
ENSMUSG00000024248	cytochrome c oxidase subunit 7A2 like (Cox7a2l)
ENSMUSG00000055302	Morf4 family associated protein 1 (Mrfap1)
ENSMUSG00000031622	transcriptional regulator, SIN3B (yeast) (Sin3b)
ENSMUSG00000027422	ribosome binding protein 1 (Rrbp1)
ENSMUSG00000020232	high mobility group 20B (Hmg20b)
ENSMUSG00000020300	cytoplasmic polyadenylation element binding protein 4 (Cpeb4)
ENSMUSG00000021714	centromere protein K (Cenpk)
ENSMUSG00000028797	transmembrane protein 234 (Tmem234)
ENSMUSG00000095186	
ENSMUSG00000096474	
ENSMUSG00000096201	
ENSMUSG00000081671	
ENSMUSG00000095865	
ENSMUSG00000053706	
ENSMUSG00000108465	
ENSMUSG00000092595	
ENSMUSG00000091028	
ENSMUSG00000091275	
ENSMUSG00000112308	
ENSMUSG00000083929	
ENSMUSG00000105341	
ENSMUSG00000091623	
ENSMUSG00000082896	
ENSMUSG00000091542	
ENSMUSG00000112947	
ENSMUSG00000095464	
ENSMUSG00000112808	
ENSMUSG00000095891	
ENSMUSG00000112336	
ENSMUSG00000096850	
ENSMUSG00000090338	
ENSMUSG00000114934	
ENSMUSG00000110711	
ENSMUSG00000096385	
ENSMUSG00000095562	

Gene ID	Name
ENSMUSG00000045799	
ENSMUSG00000094526	
ENSMUSG00000084279	
ENSMUSG00000114763	
ENSMUSG00000096768	
ENSMUSG00000100954	
ENSMUSG00000094293	
ENSMUSG00000073879	
ENSMUSG00000113811	
ENSMUSG00000085178	
ENSMUSG00000112852	
ENSMUSG00000095547	
ENSMUSG00000110411	

Dataset S3. List of m^6A-methylation targets in mouse embryonic hearts.

Name	Gene ID	Diff. log_2 (Fold change)	*P*-value
Adamts1	ENSMUSG00000022893	-0.5872	0.0035
Speg	ENSMUSG00000026207	-0.8090	0.0000
Bmpr1a	ENSMUSG00000021796	-0.8883	0.0046
Cacna1c	ENSMUSG00000051331	-0.5154	0.0055
Vcan	ENSMUSG00000021614	-0.6510	0.0049
Gata5	ENSMUSG00000015627	-0.6726	0.0075
Gja1	ENSMUSG00000050953	-0.4121	0.0001
Hdac5	ENSMUSG00000008855	-1.0173	0.0012
		-0.8011	0.0083
Kdr	ENSMUSG00000062960	-0.5690	0.0096
Mef2a	ENSMUSG00000030557	-0.3436	0.0072
Pkd2	ENSMUSG00000034462	-0.6240	0.0082
Plec	ENSMUSG00000022565	-1.0201	0.0058
Sema3c	ENSMUSG00000028780	-0.4094	0.0021
Stil	ENSMUSG00000028718	-1.5422	0.0100
Sin3b	ENSMUSG00000031622	-1.4778	0.0014
Slc8a1	ENSMUSG00000054640	-0.4743	0.0002
		-0.7049	0.0000
Sox11	ENSMUSG00000063632	-1.5027	0.0017
Sox4	ENSMUSG00000076431	-0.5208	0.0000
		-0.5251	0.0044
Tgfb2	ENSMUSG00000039239	-1.0703	0.0030
Ttn	ENSMUSG00000051747	-1.6498	0.0002
Twist1	ENSMUSG00000035799	-0.5734	0.0011
Cntrl	ENSMUSG00000057110	-0.6938	0.0090
Popdc2	ENSMUSG00000022803	-0.5283	0.0000
Ift74	ENSMUSG00000028576	-0.5972	0.0023
Jmjd6	ENSMUSG00000056962	-0.9836	0.0077
Alpk3	ENSMUSG00000038763	-0.7700	0.0018
Myocd	ENSMUSG00000020542	-0.8312	0.0001
Kdm6b	ENSMUSG00000018476	-0.6866	0.0008

Dataset S4. Phenotypes of the hypomethylated candidate gene knockout mice.

Name	PMID	Gene-editing mouse	Lethality	Cardiovascular phenotypes/abnormalities
Adamts1	18267097	$Adamts1^{-/-}$	approximately 50% embryonic lethality	hypertrabeculation in the ventricle
Speg	26593099	$Speg^{-/-}$	a significant amount of in utero death	dilated cardiomyopathy
Bmpr1a	18667463	SM22α-Cre; R26R; $Bmpr1a^{flox/flox}$	embryonic lethality after E11.0	massive vascular and pericardial hemorrhage, thinning of the myocardium
Cacna1c	21832054	$Cav1.2^{D1904Stop/D1904Stop}$	died after birth	normal septum thickness, heart failure
Vcan	22692047	$Vcan^{\Delta3/\Delta3}$	died during the early development stage	narrow cardiac jelly, smaller atrioventricular canal cushion
Gata5	21633169	$Gata5^{-/-}$	N/A	thinning ventricular wall, bicuspid aortic valve
Gja1	7892609	$Cx43^{-/-}$	perinatal mortality	blockage of the right ventricular outflow tract
Hdac5	15367668	$Hdac5^{-/-}$	N/A	age-dependent cardiac hypertrophy
Kdr	38618720	$VEGFR2^{flox/-}$; Mef2C-AHF-Cre+; $Rosa^{tdTom/+}$	embryonic lethality shortly after mid-gestation	pharyngeal arch artery defects
Mef2a	12379849	$Mef2a^{-/-}$	postnatal lethality	right ventricular hypoplasia and chamber dilation
Pkd2	10615132	$Pkd2^{-/-}$	die in utero between E13.5 and parturition	ventricular septal defect, atrial septal defect
Plec	9389647	$Plec^{-/-}$	die within a few days after birth	disintegration of intercalated discs in the heart
Sema3c	11688556	$Sema3C^{-/-}$	postnatal mortality	persistent truncus arteriosus, interruption of the aortic arch
Stil	10385121	$Stil^{-/-}$	die at mid-gestation	randomized heart looping
Sin3b	18332431	$Sin3b^{-/-}$	late stage embryonic lethal	N/A
Slc8a1	11211878	$Ncx1^{-/-}$	embryonic lethality within E9.0-9.5	underdeveloped heart with a dilated pericardium
Sox11	15254231	$Sox11^{-/-}$	postnatal lethality	ventricular septal defect, outflow tract malformations
Sox4	8614465	$Sox4^{-/-}$	embryonic death at E14.0	ventricular septal defect, outflow tract malformations

Name	PMID	Gene-editing mouse	Lethality	Cardiovascular phenotypes/abnormalities
Ttn	15136139	Titin shrunken-head mutation	lethality by E11.5	weakly contractile, pericardial edema
Twist1	7729687	$Twist^{-/-}$	died at E11.5	N/A
Cntrl	N/A	N/A	N/A	N/A
Popdc2	32535041	$Popdc2^{-/-}$	N/A	stress-induced bradycardia
Ift74	37315079	$Ift74^{Tm1a}$ mutation	postnatal lethality	rare
Jmjd6	15615595	$Ptdsr^{-/-}$	perinatal lethality	ventricular septal defects, double-outlet right ventricle, hypoplasia of the pulmonary artery
Alpk3	21441111	$Alpk3^{-/-}$	N/A	hypertrophic and dilated forms of cardiomyopathy
Myocd	12867591	$Myocd^{-/-}$	embryonic lethality by E10.5	defects in vascular SMC differentiation
	22996691	Nkx2-5-Cre; $Myocd^{flox/flox}$	embryonic lethality within E13.5-14.5	thinning of the compact zone and trabecular myocardium, ventricular septal defect
Kdm6b	25079229	$Jmjd3^{-/-}$	postnatal lethality	N/A
		Wnt1-Cre; $Jmjd3^{flox/flox}$		
